# Supplementary figures and images for: Comprehensive transcriptional analysis of pig facial skin development
Source: PeerJ. 2023 Aug 28;11:e15955. doi: 10.7717/peerj.15955 (PMC10470455; doi:10.7717/peerj.15955)

A

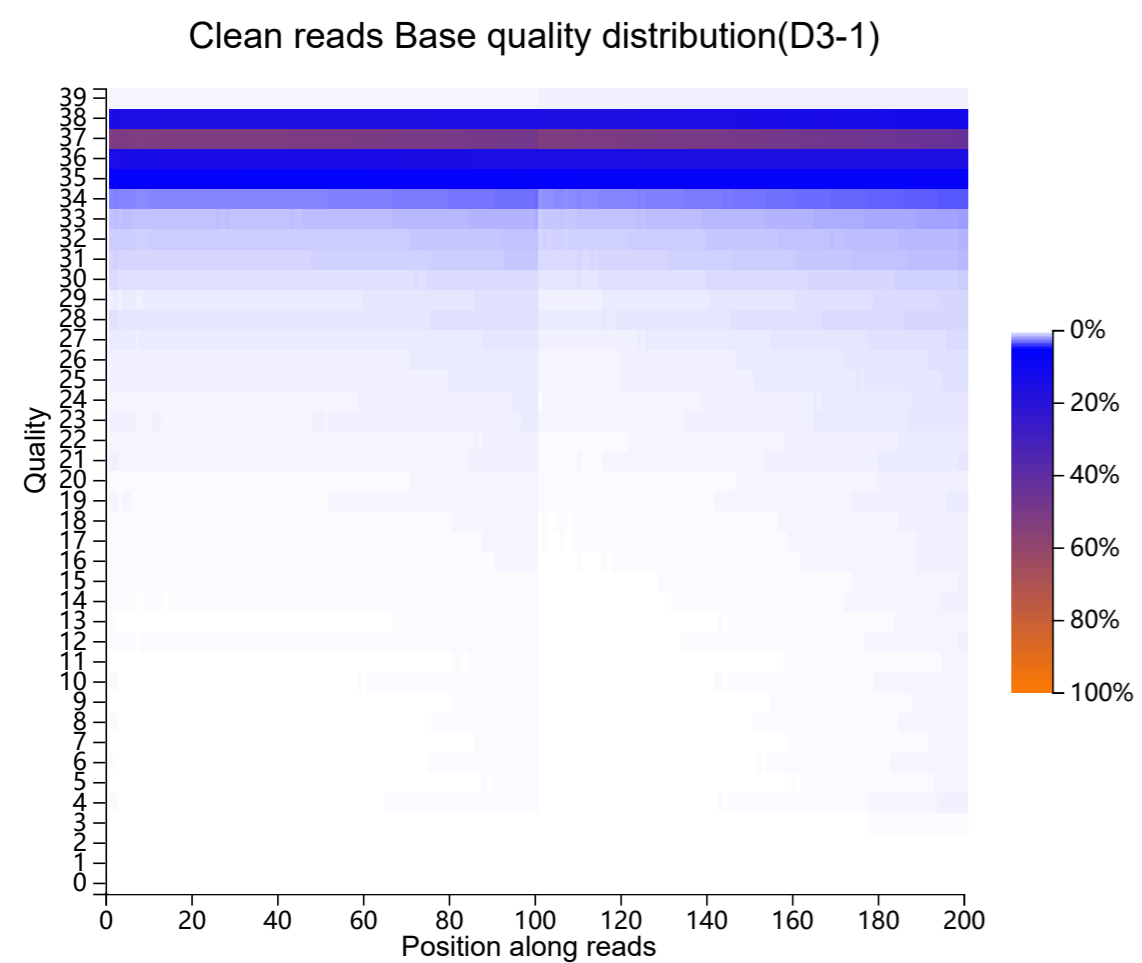

B

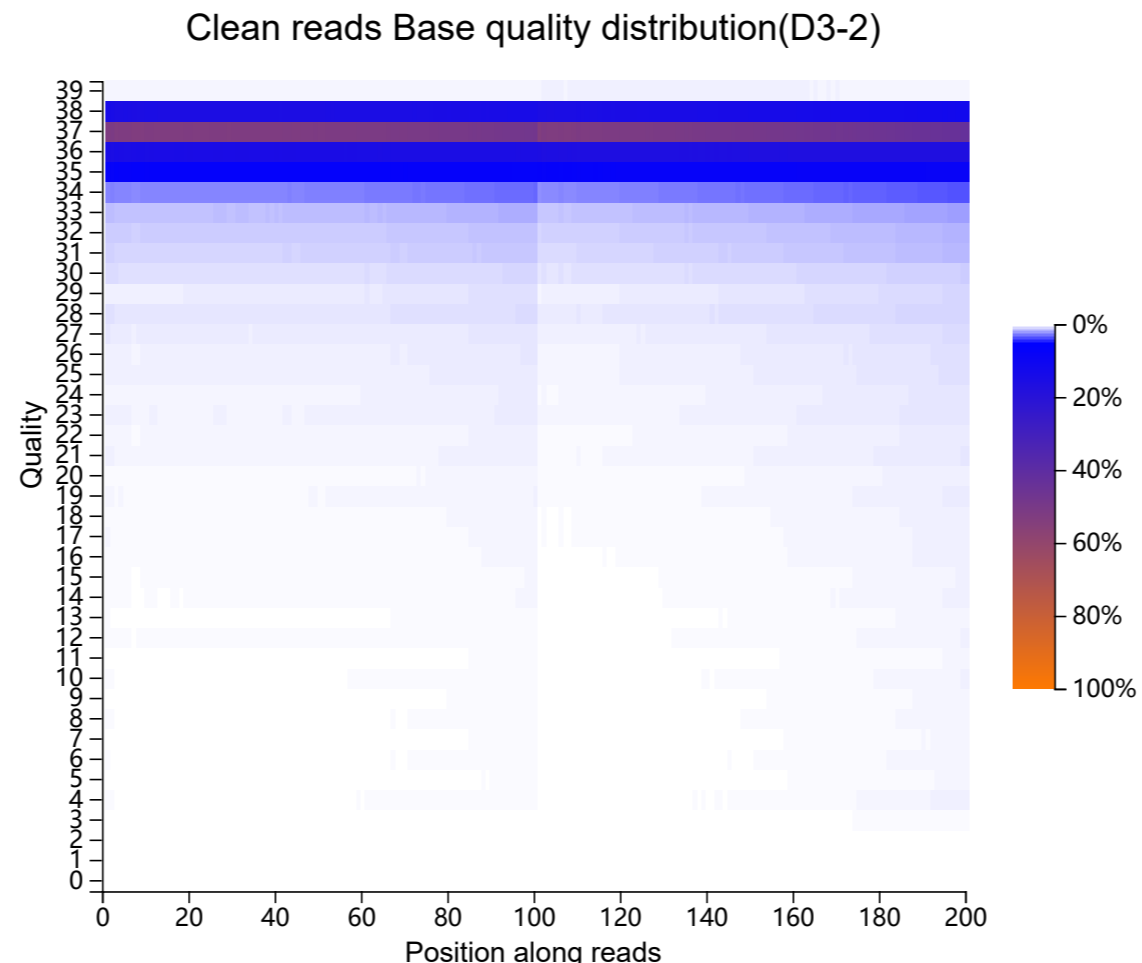

C

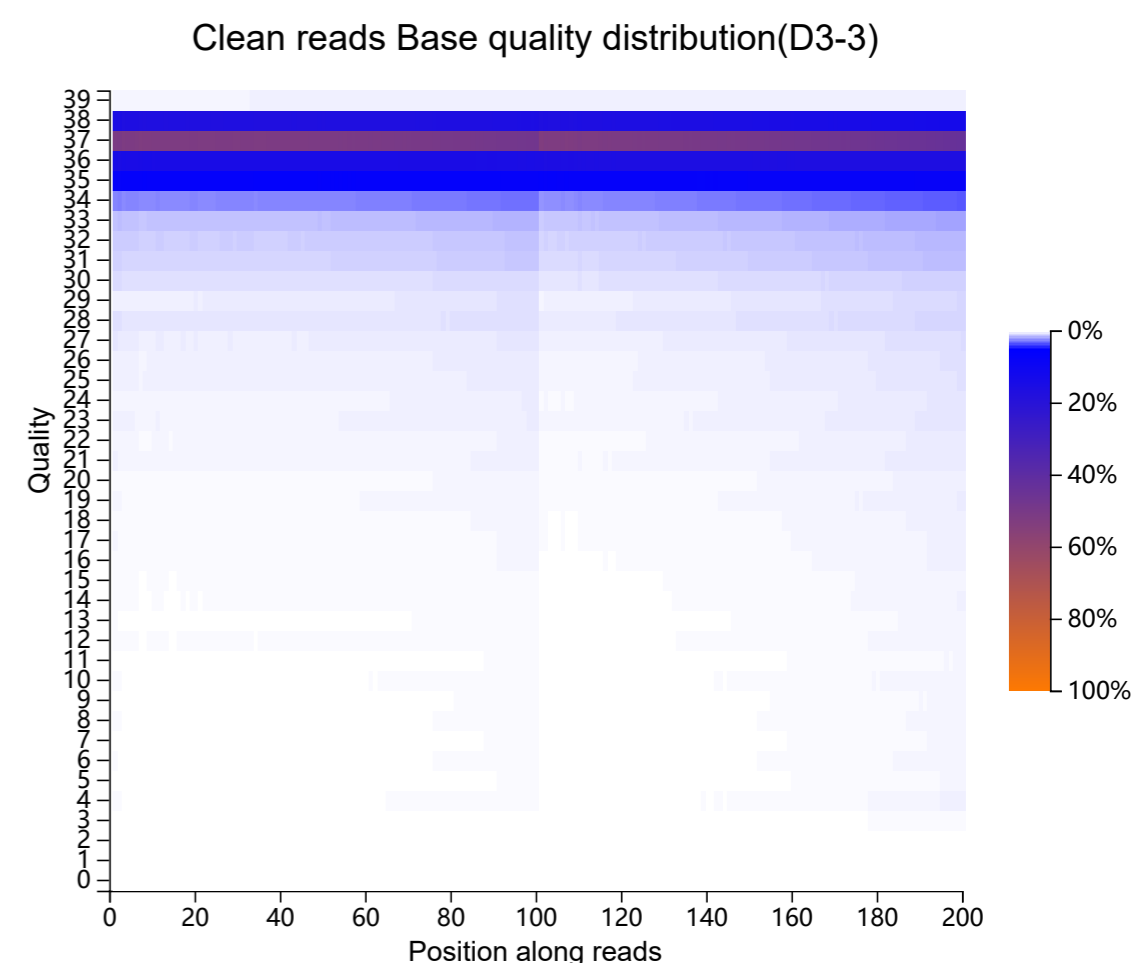

D

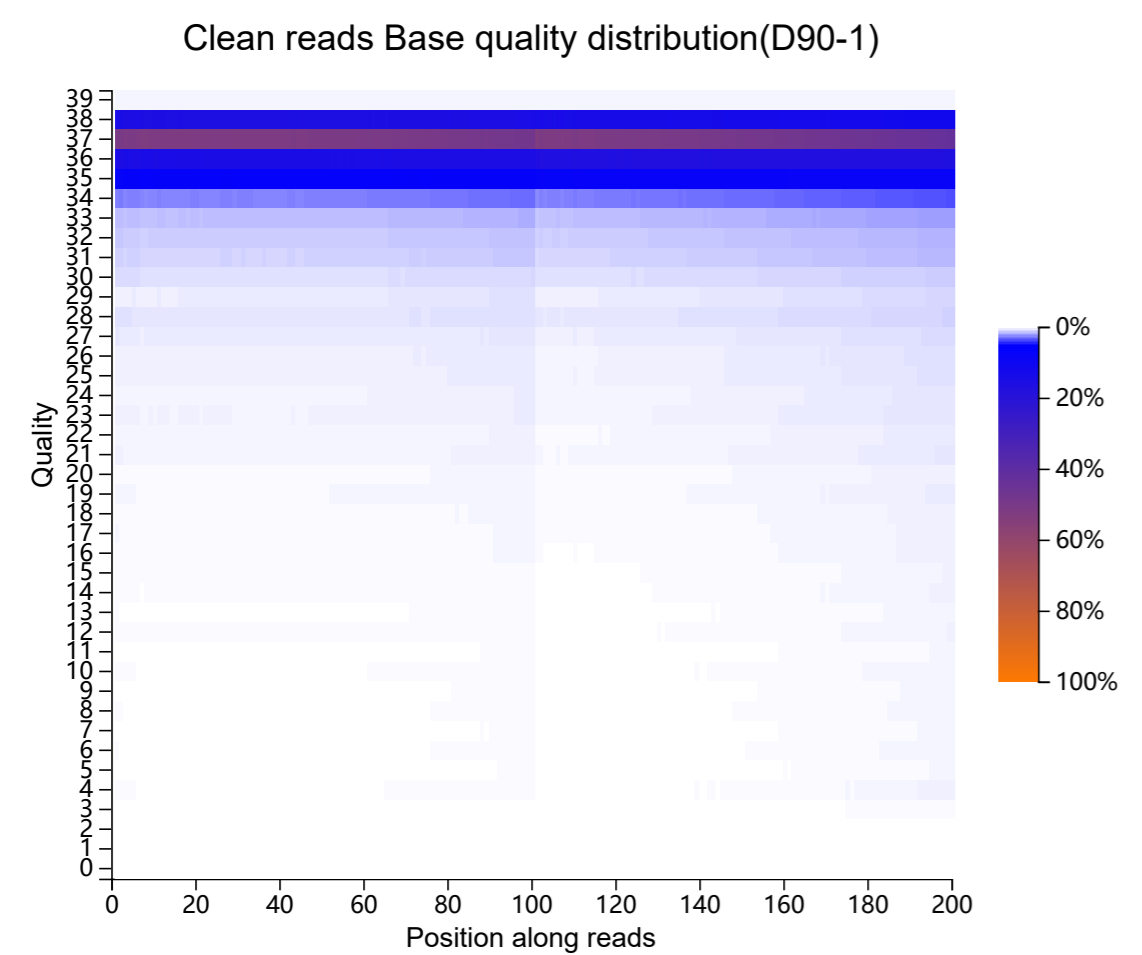

E

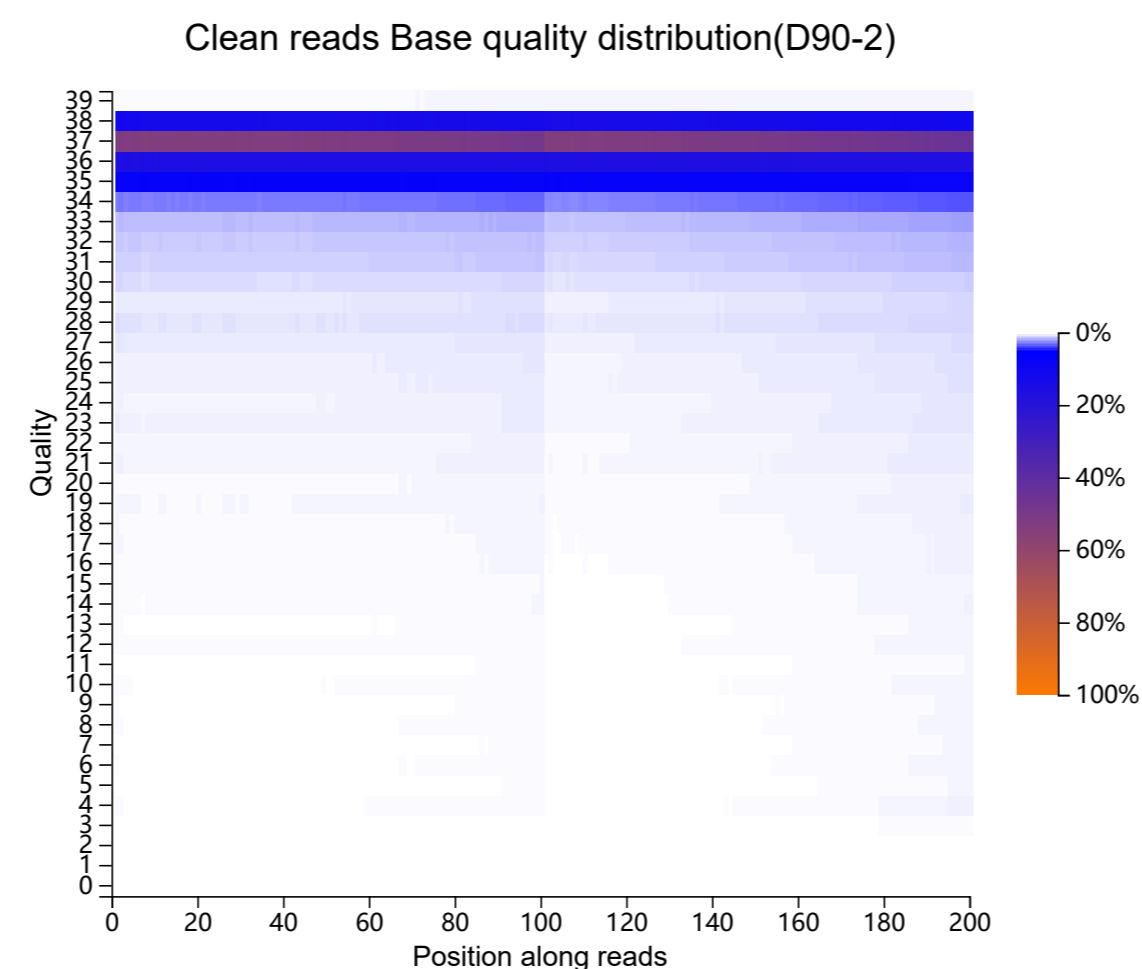

F

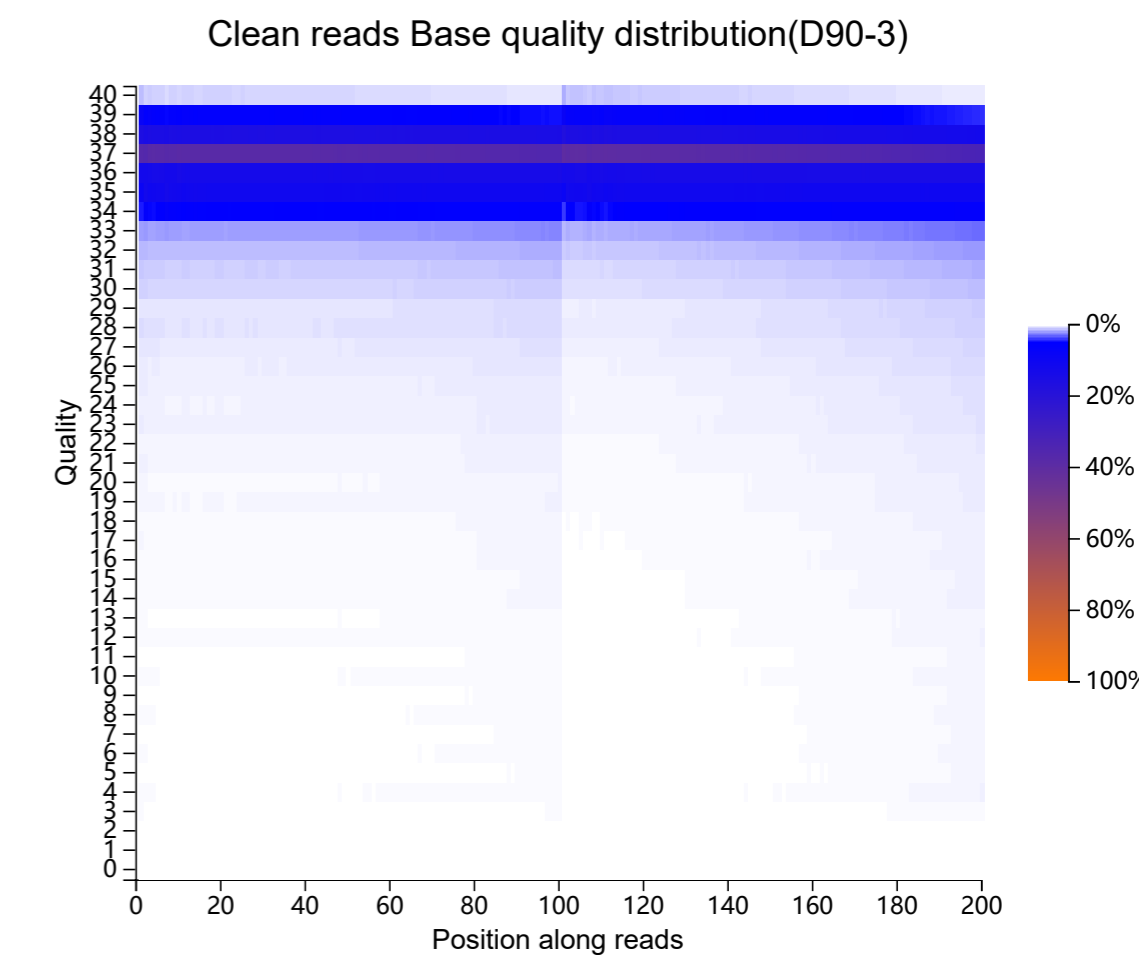

G

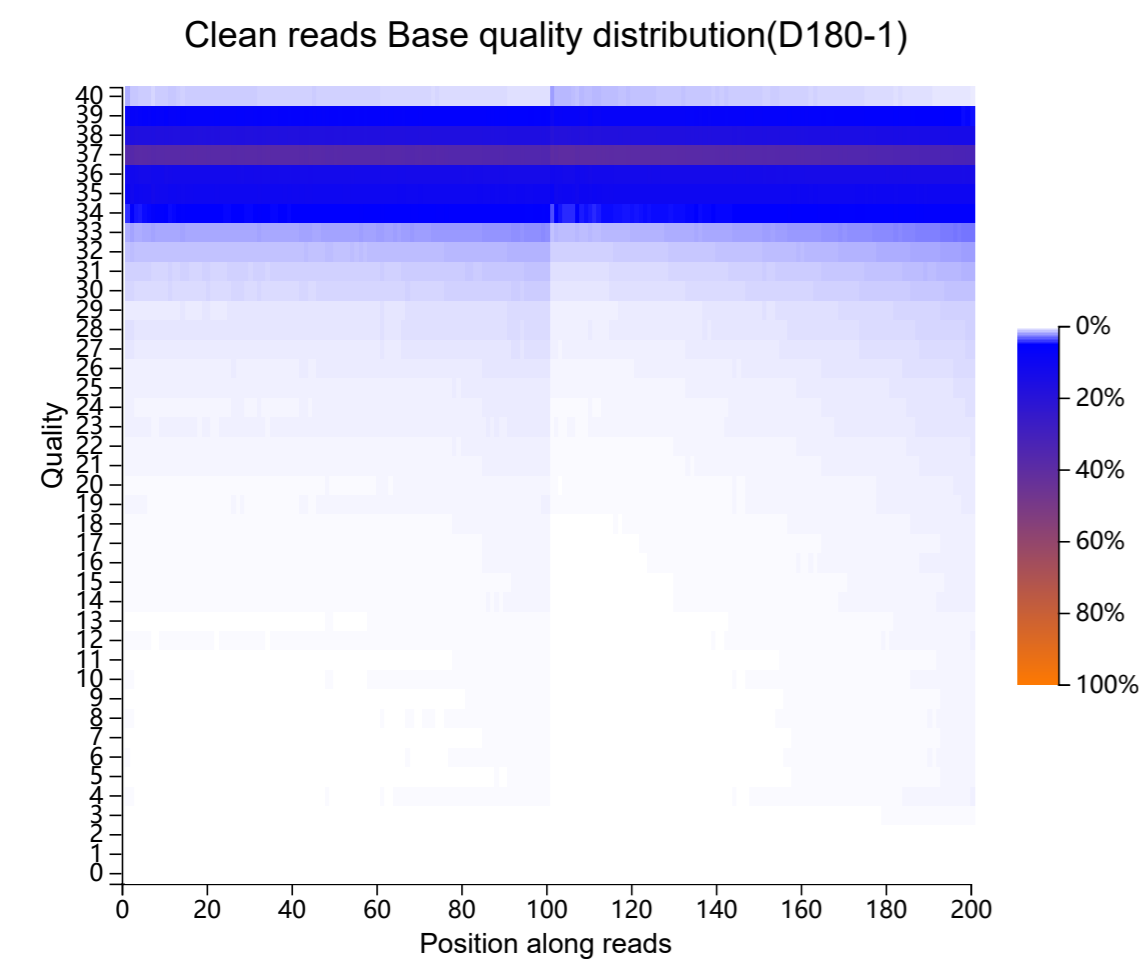

H

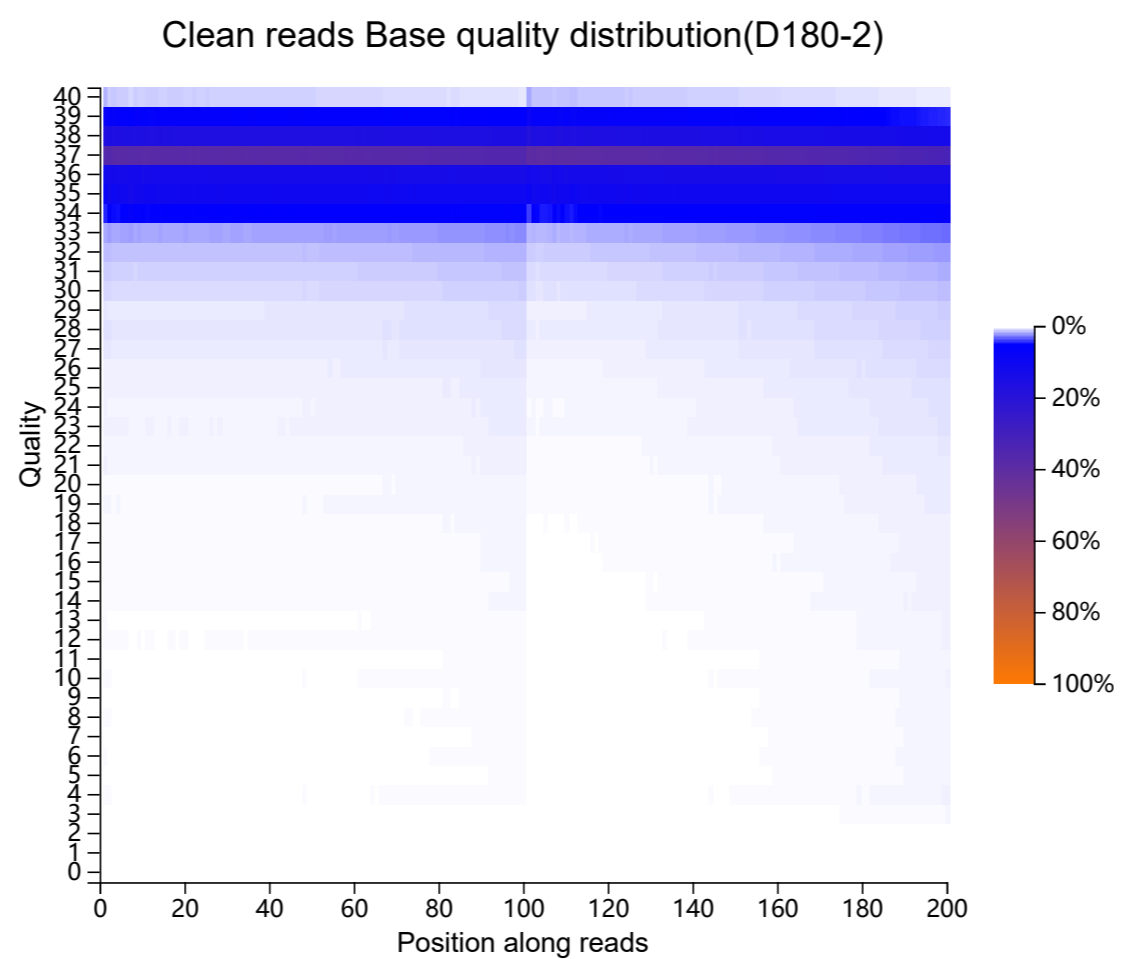

I

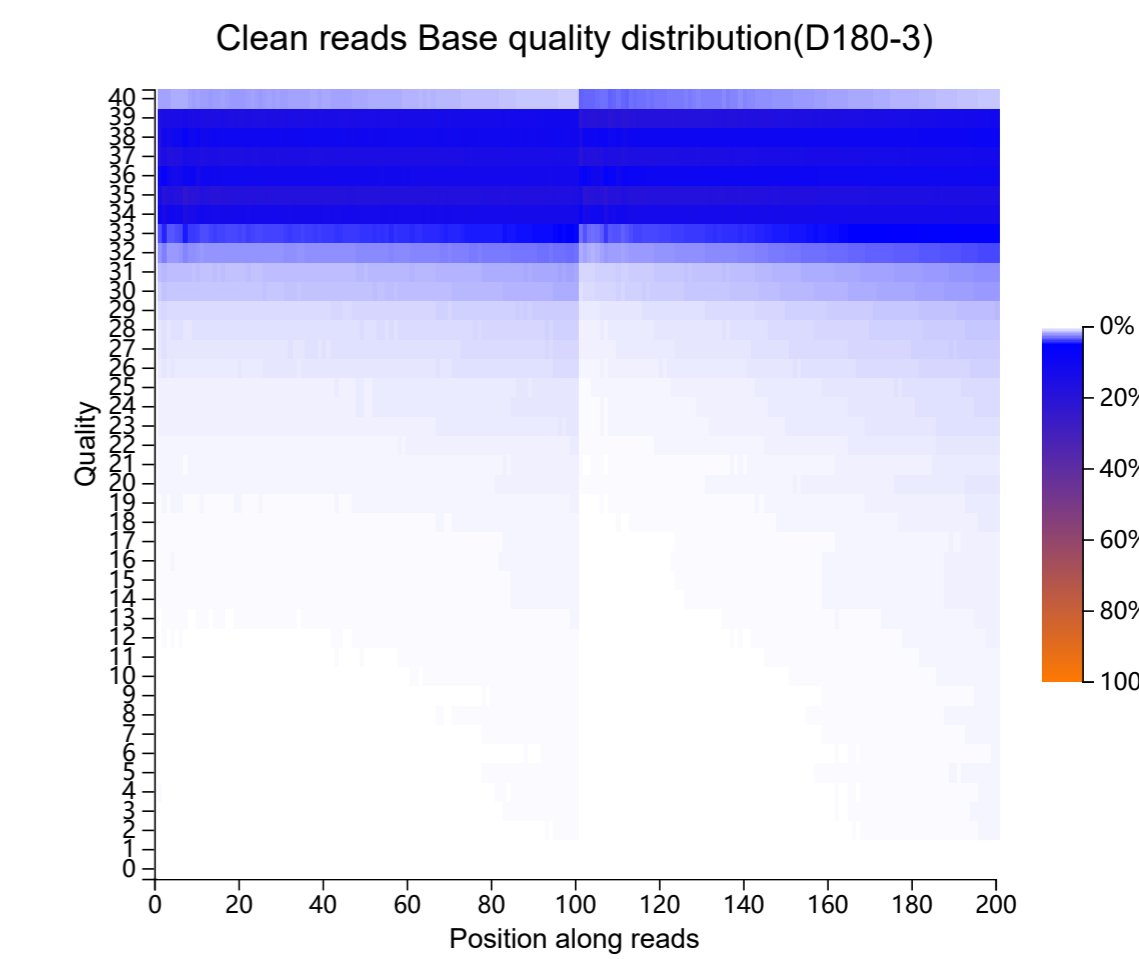

J

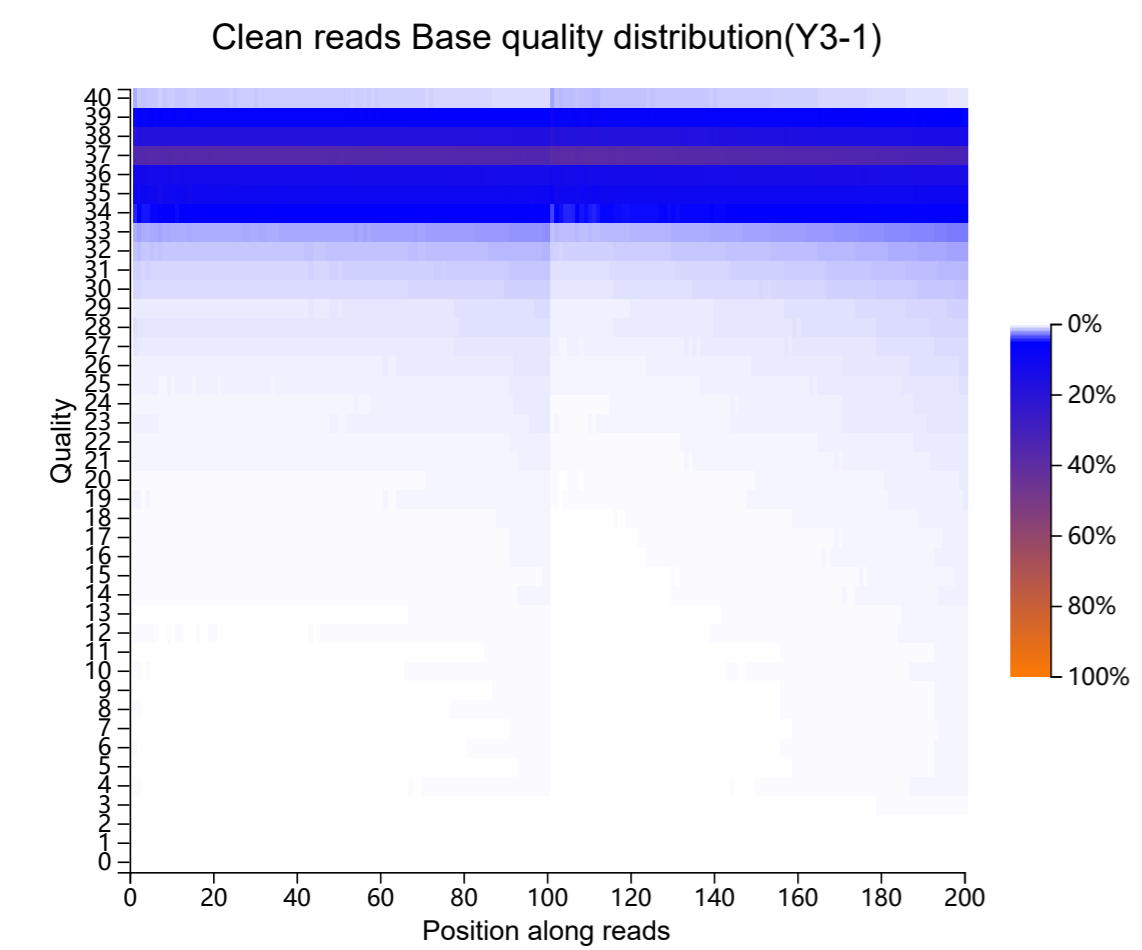

K

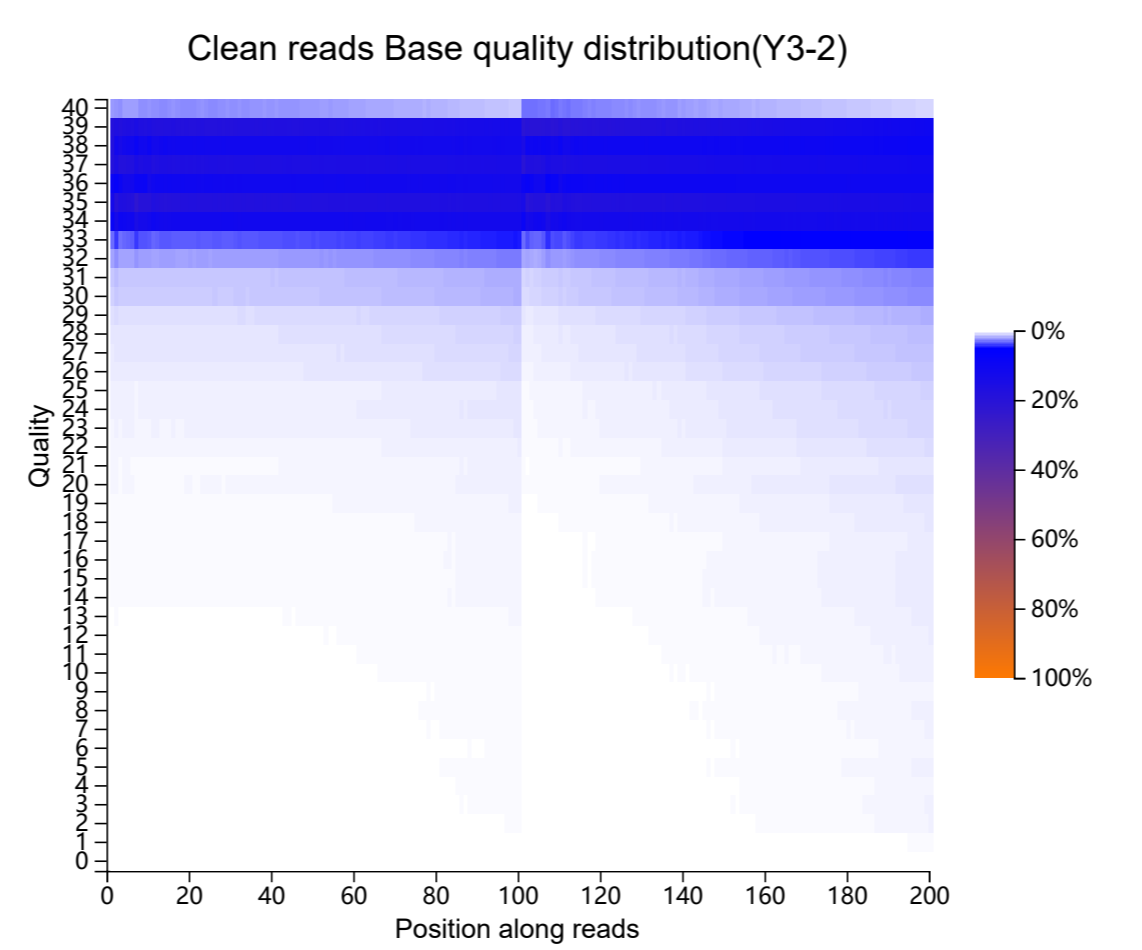

L

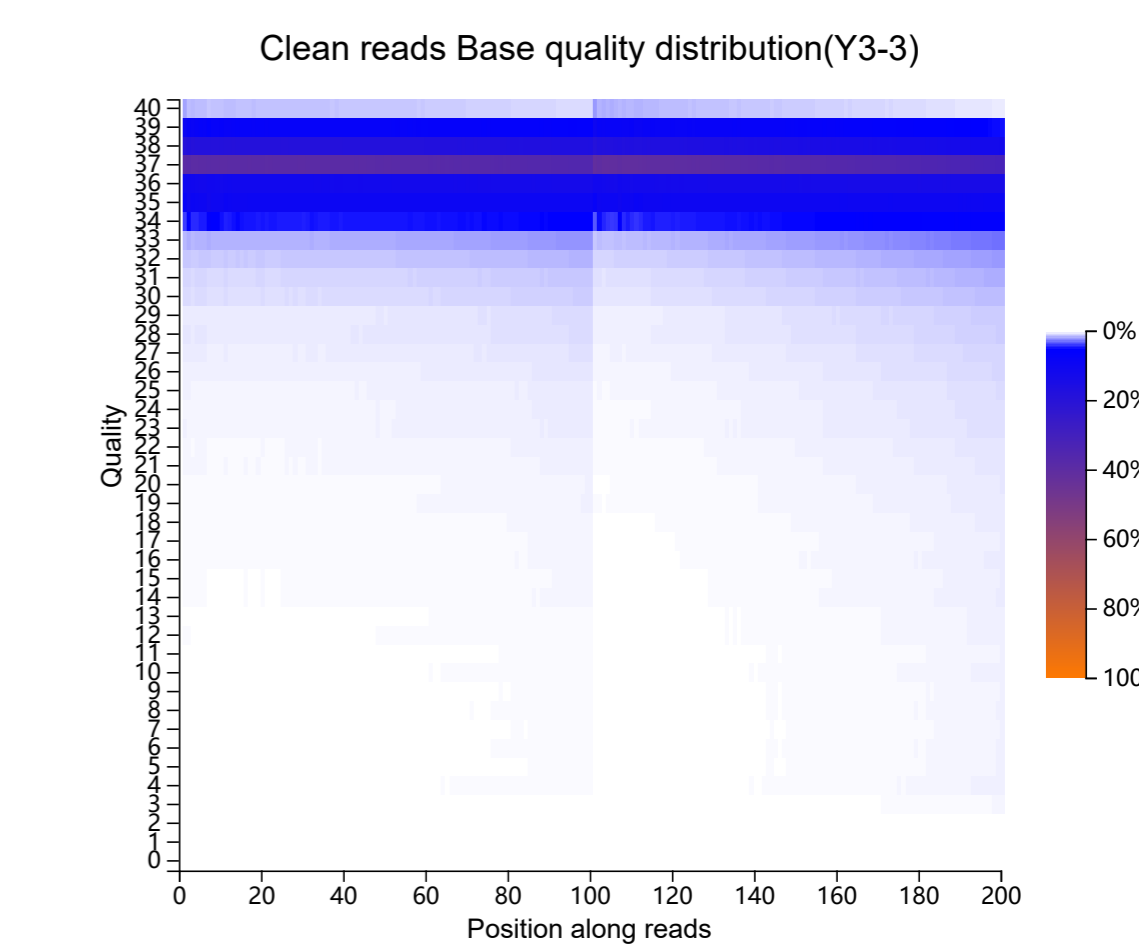

Supplement: Supplemental Information 1 [file peerj-11-15955-s001.pdf]

A

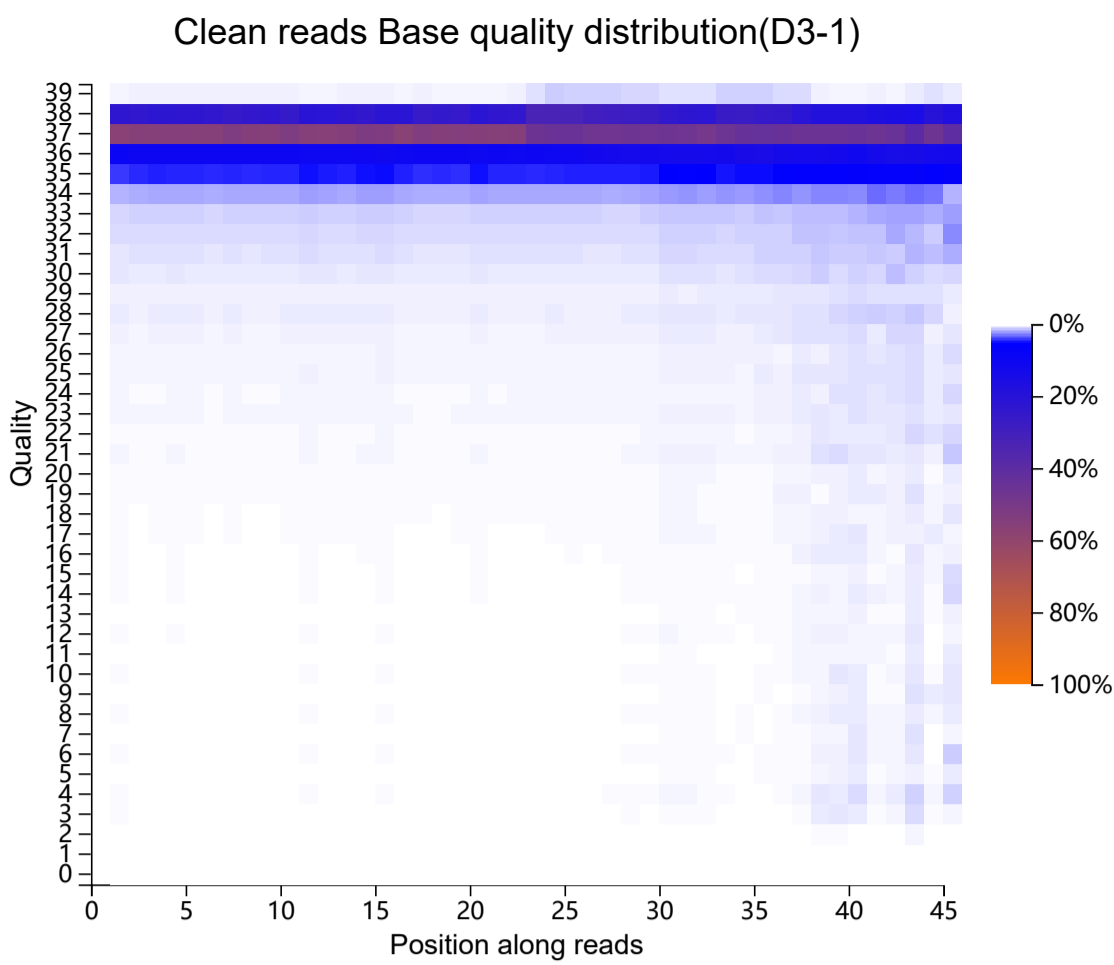

B

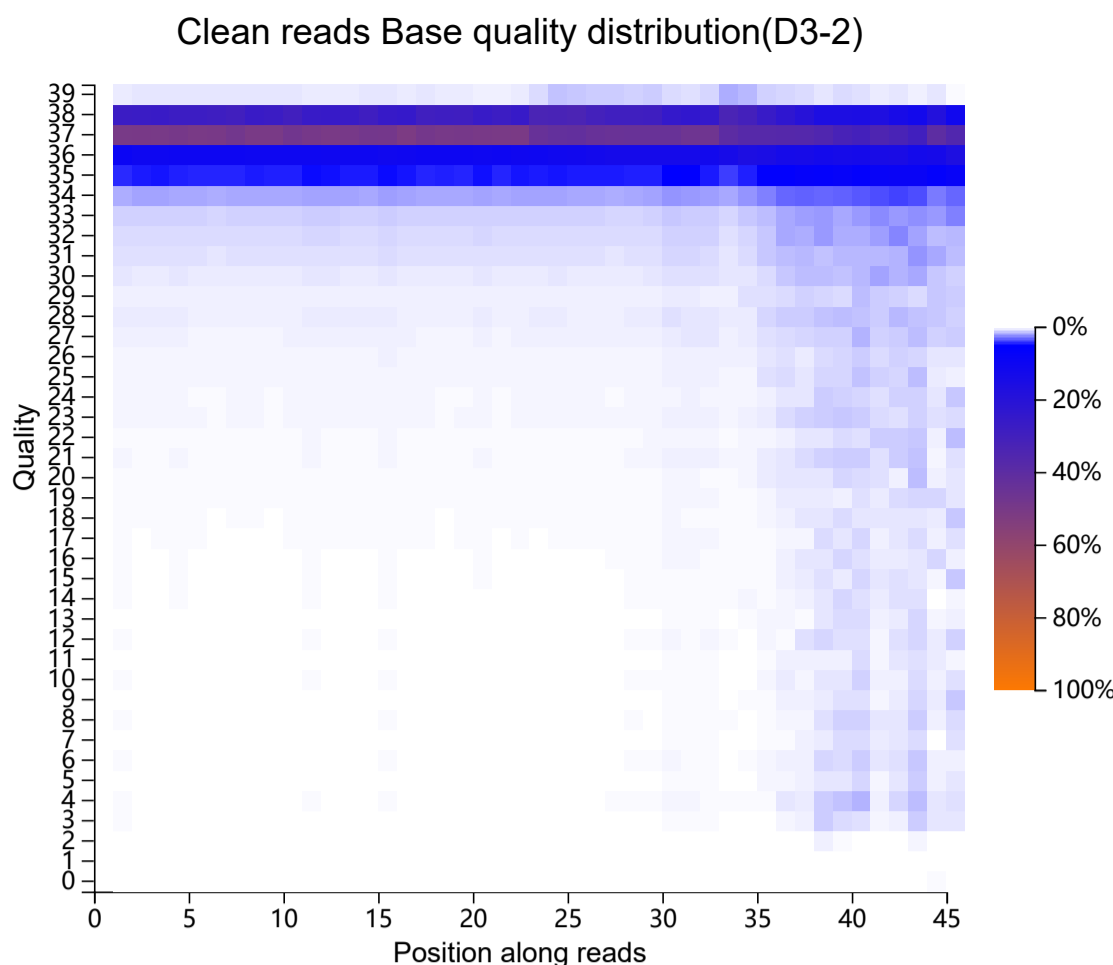

C

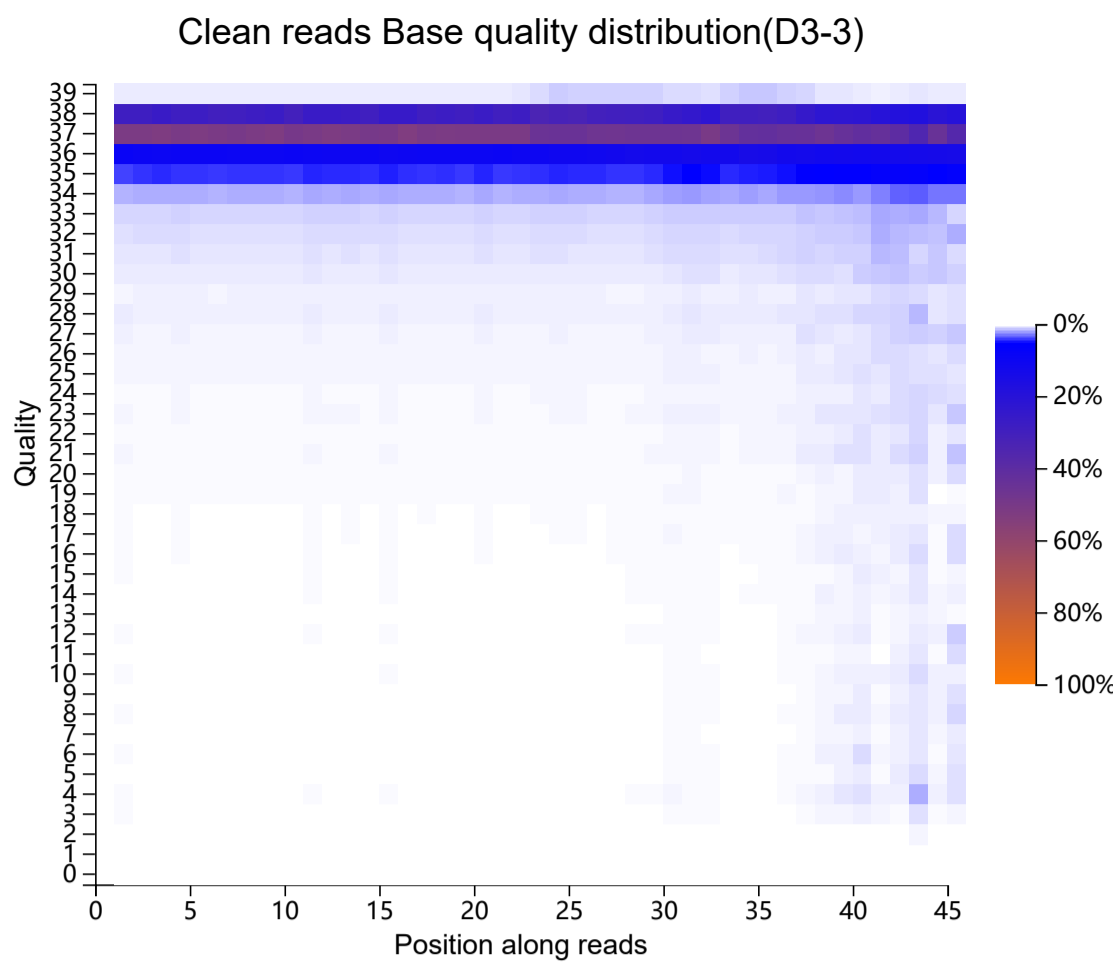

D

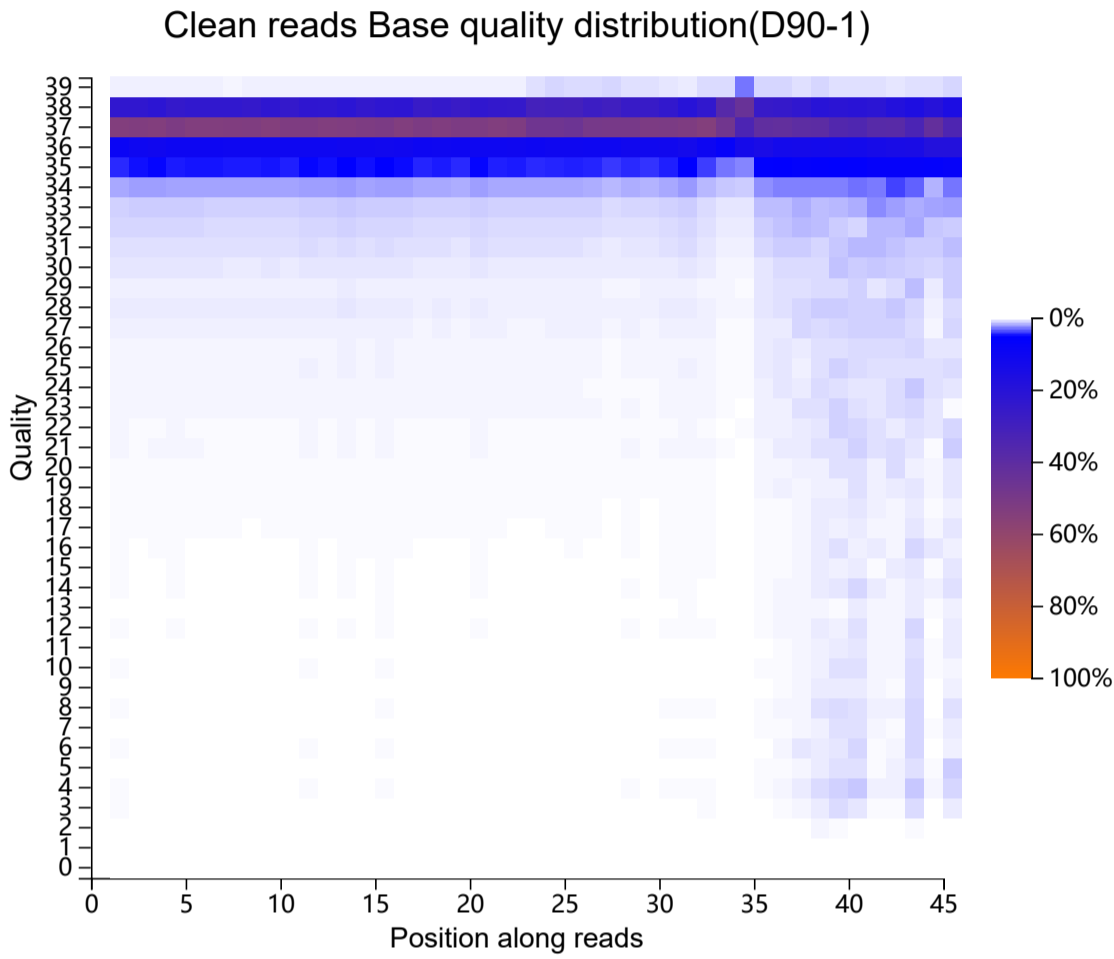

E

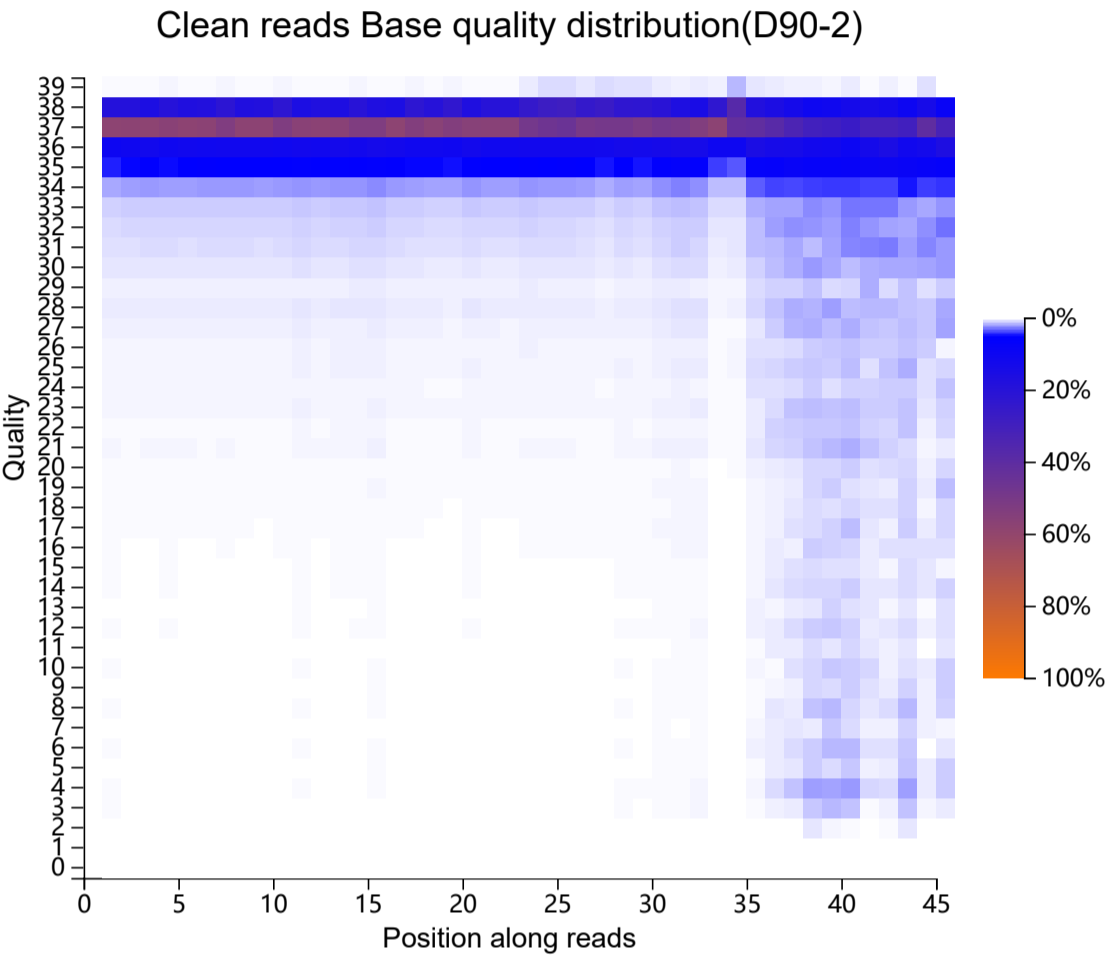

F

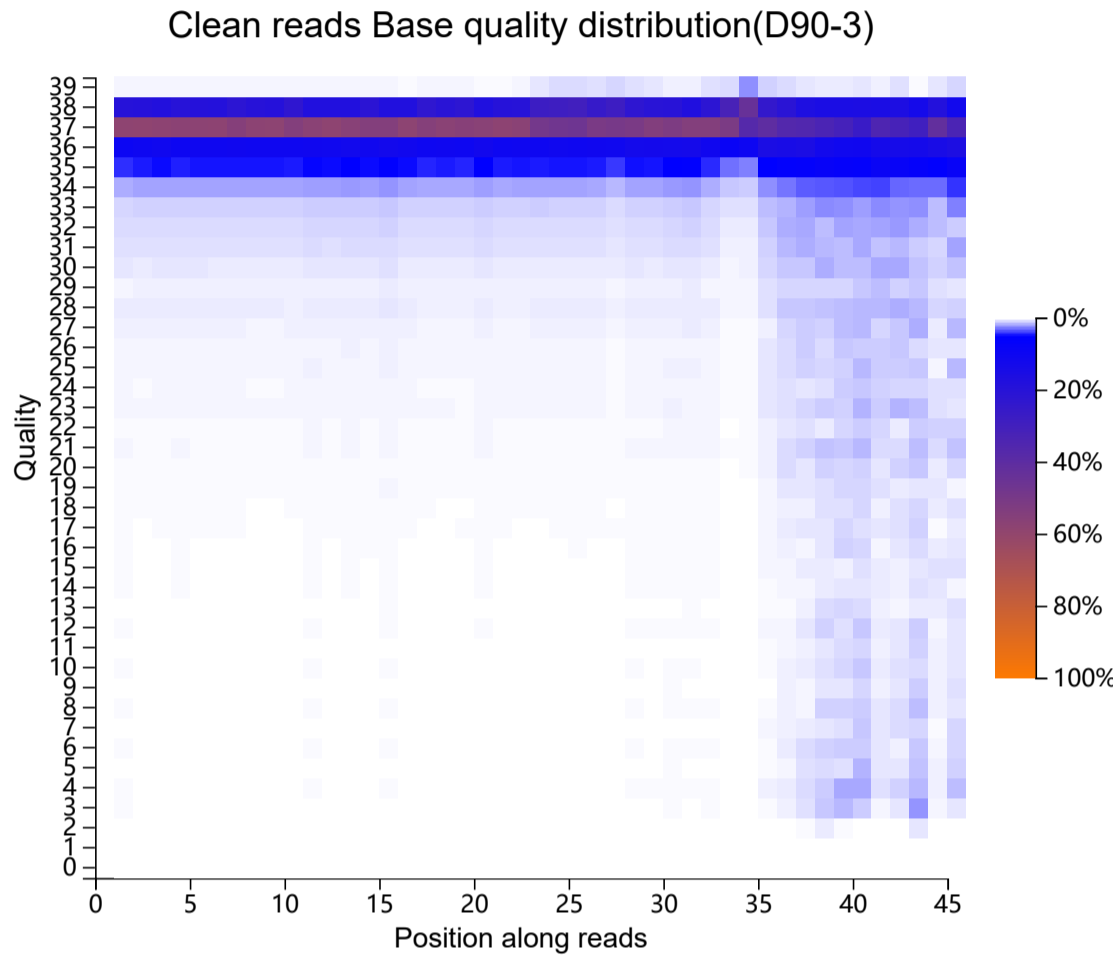

G

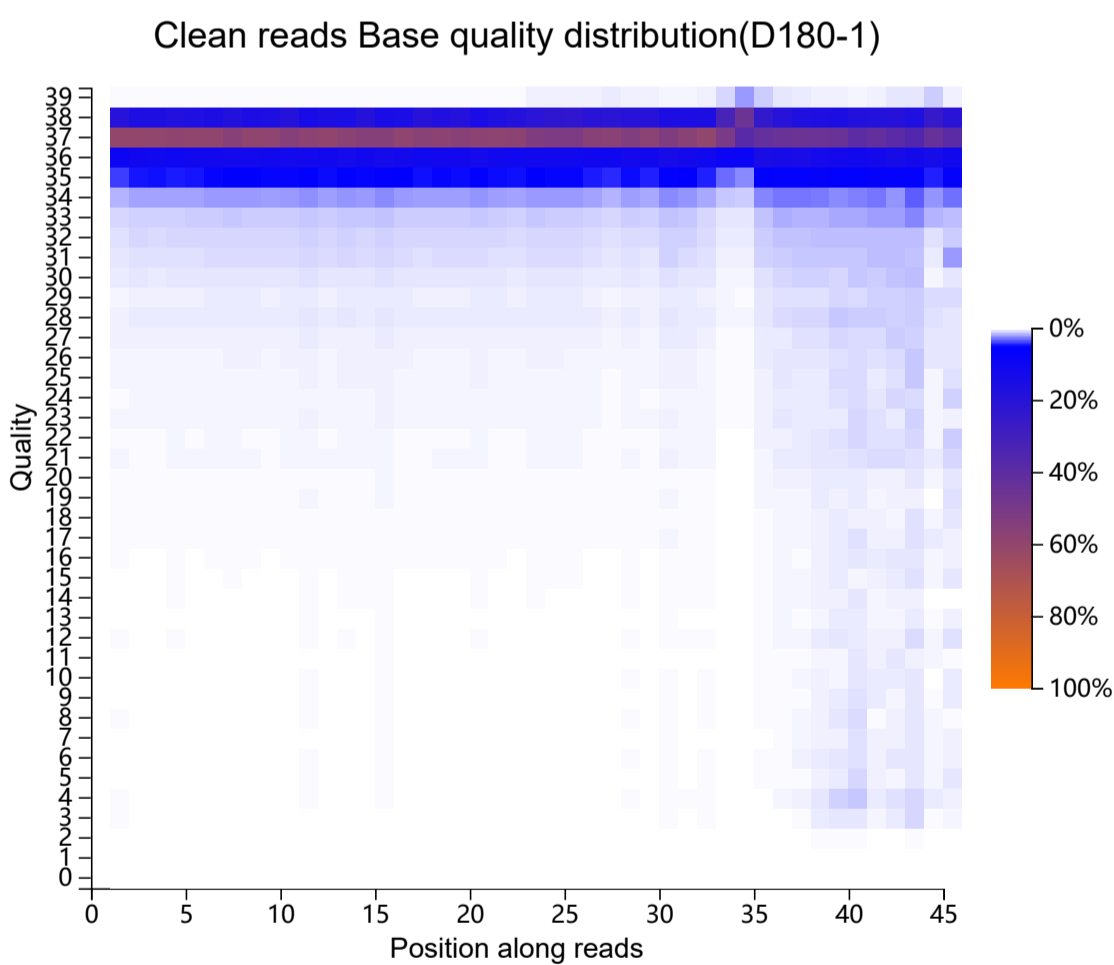

H

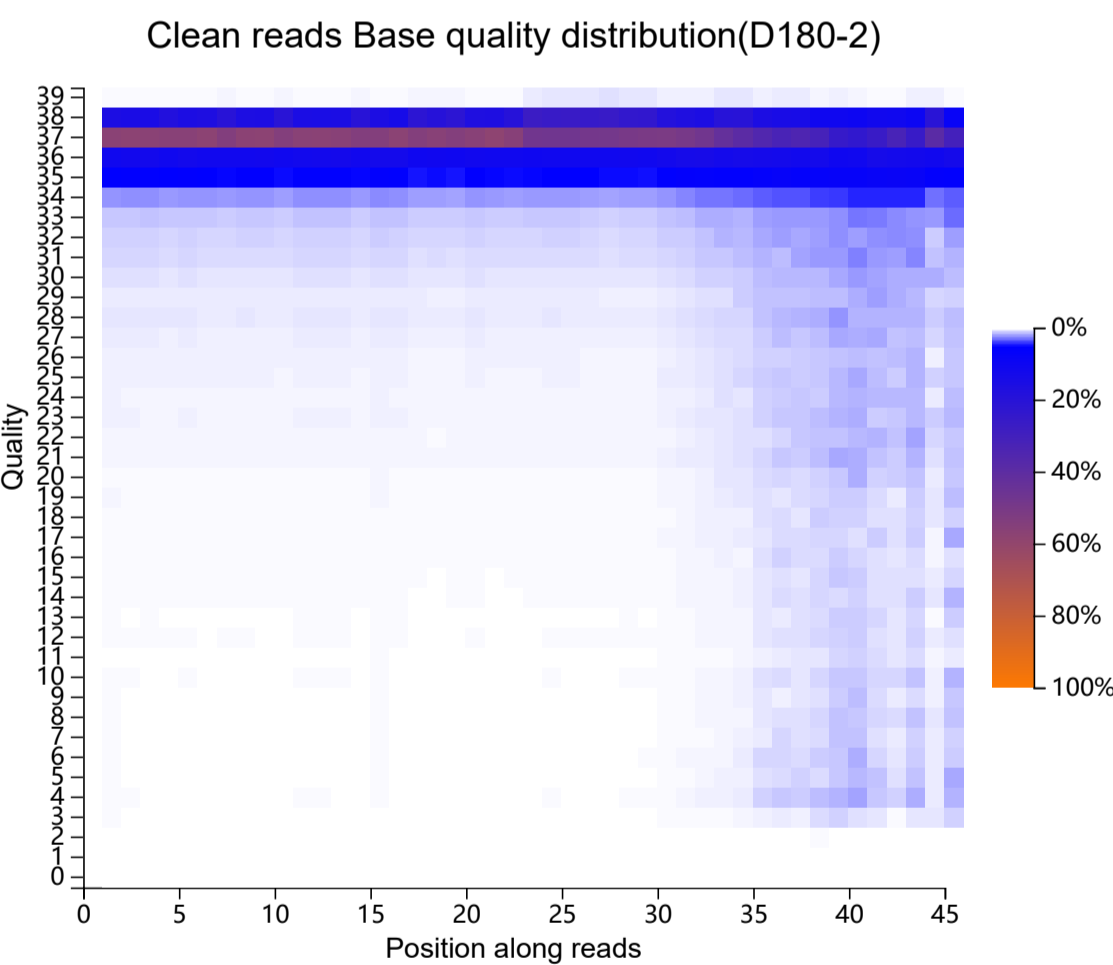

I

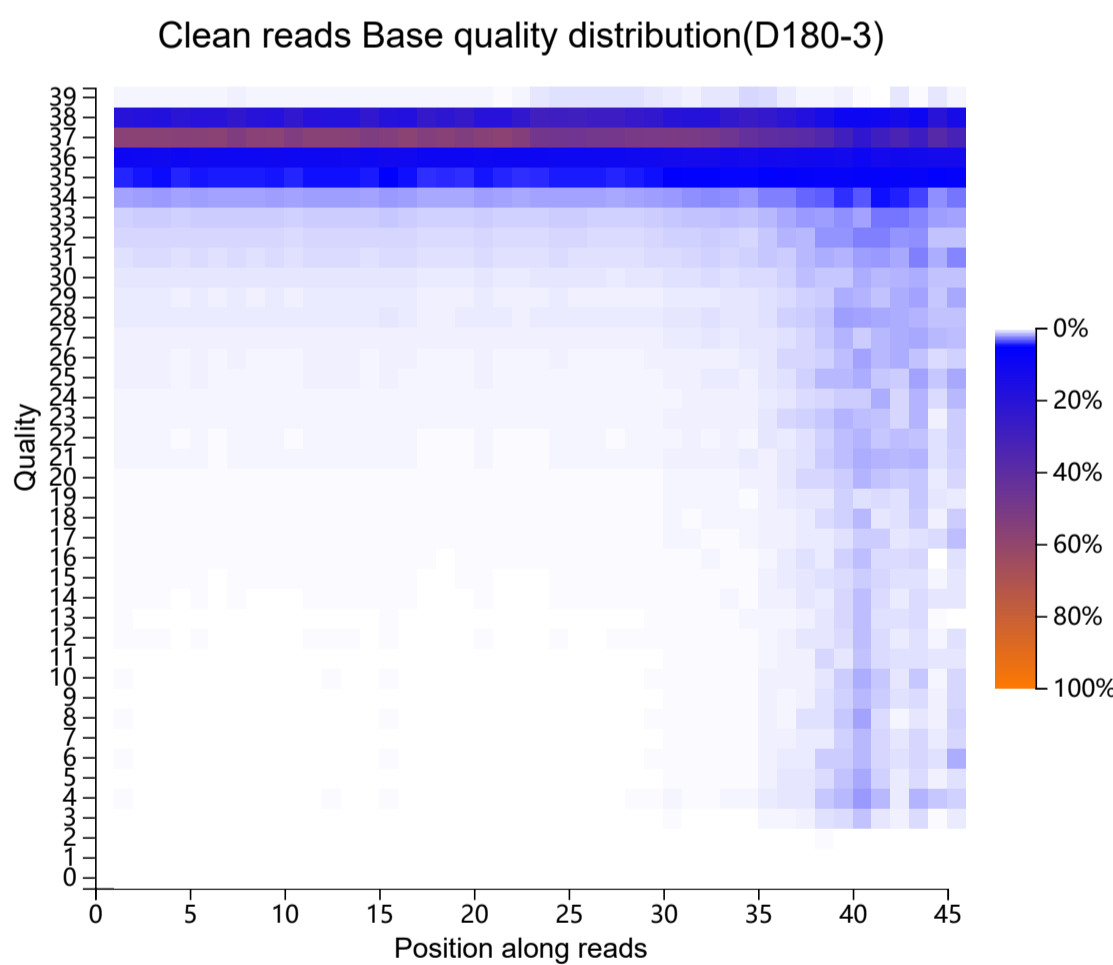

J

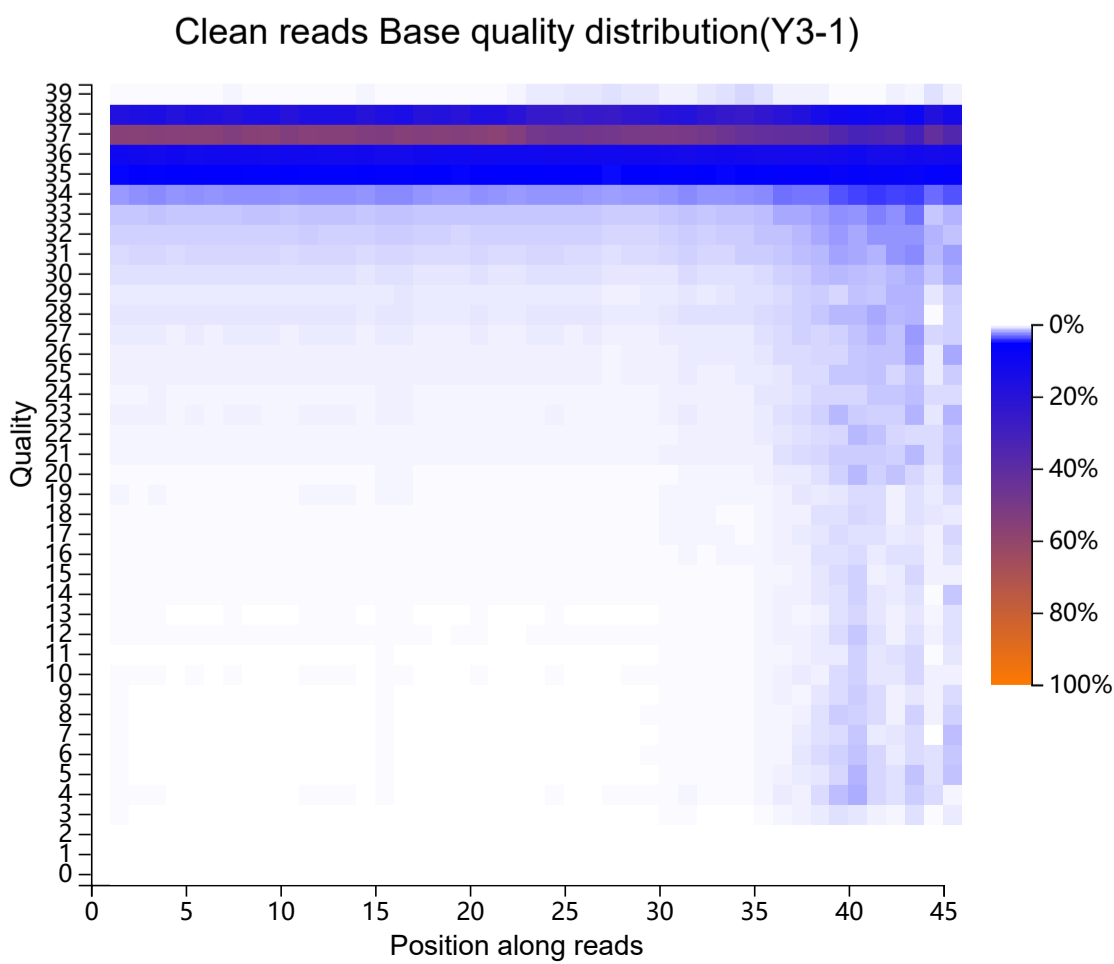

K

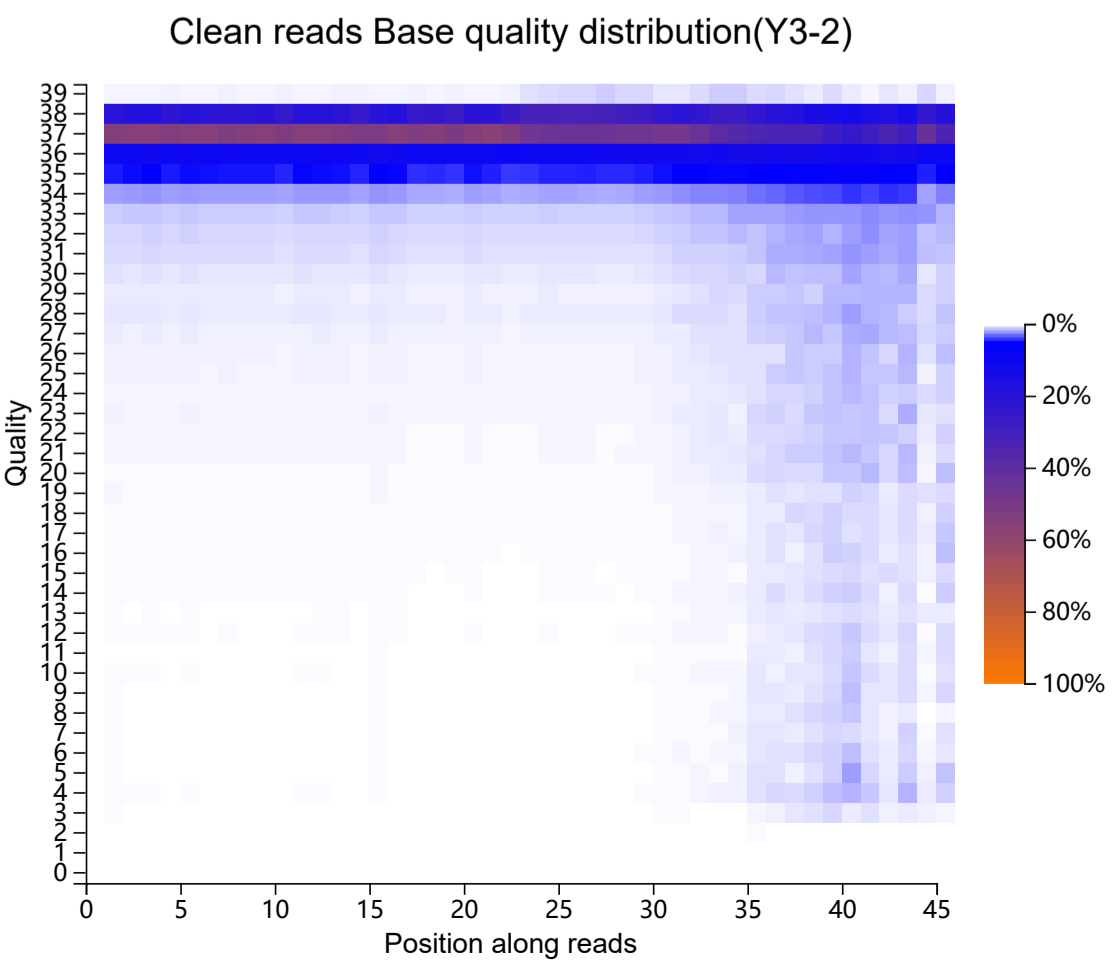

L

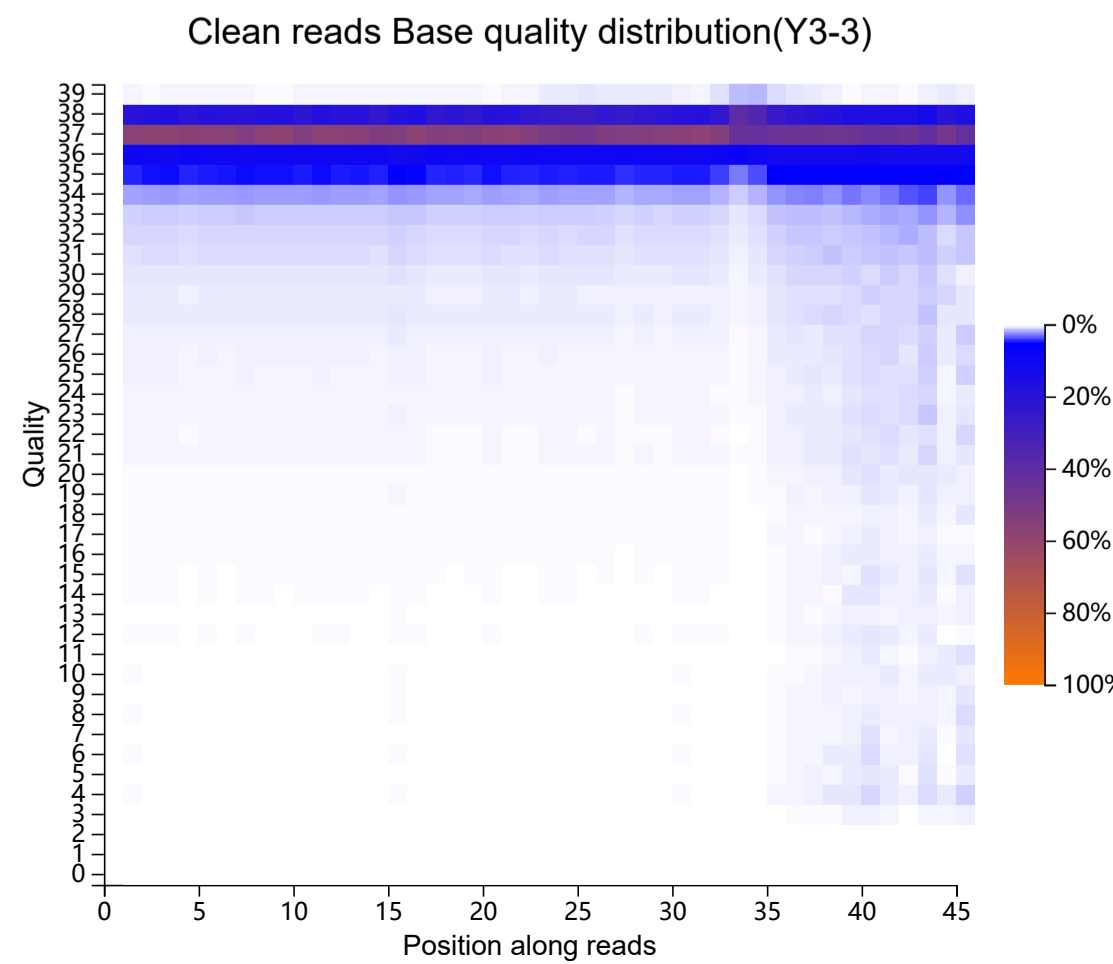

Supplement: Supplemental Information 2 [file peerj-11-15955-s002.pdf]

A

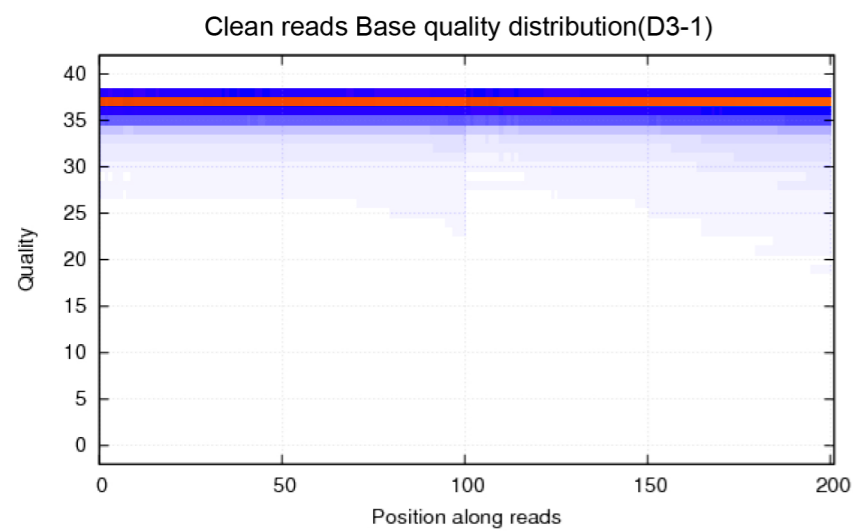

B

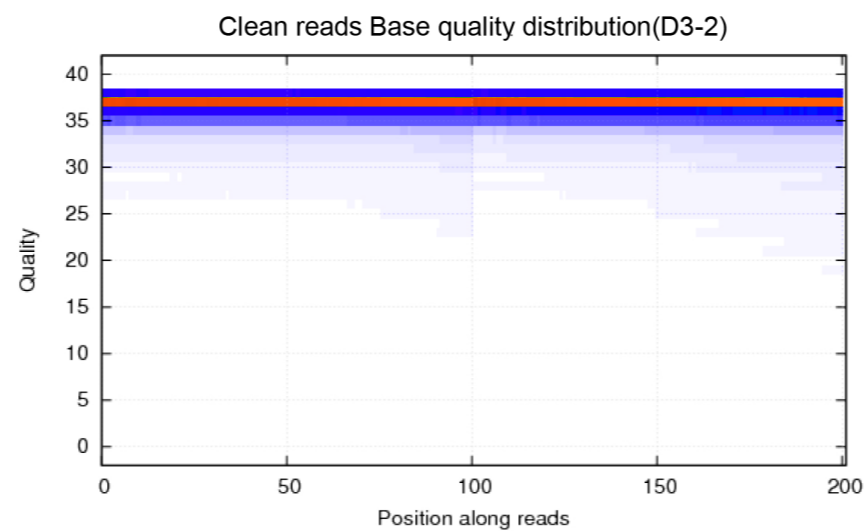

C

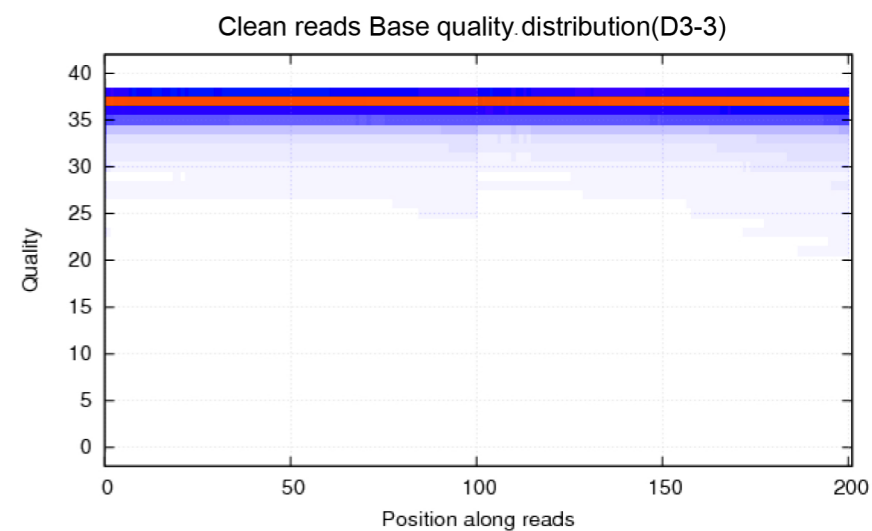

D

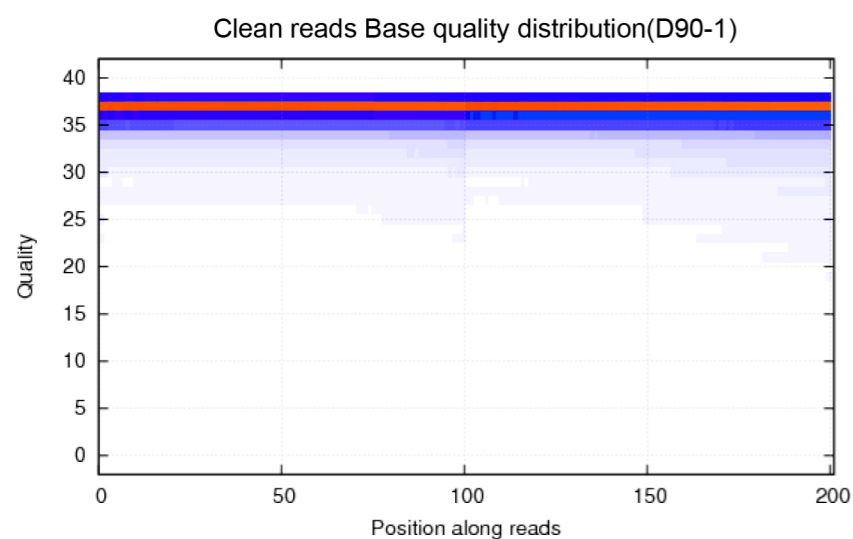

E

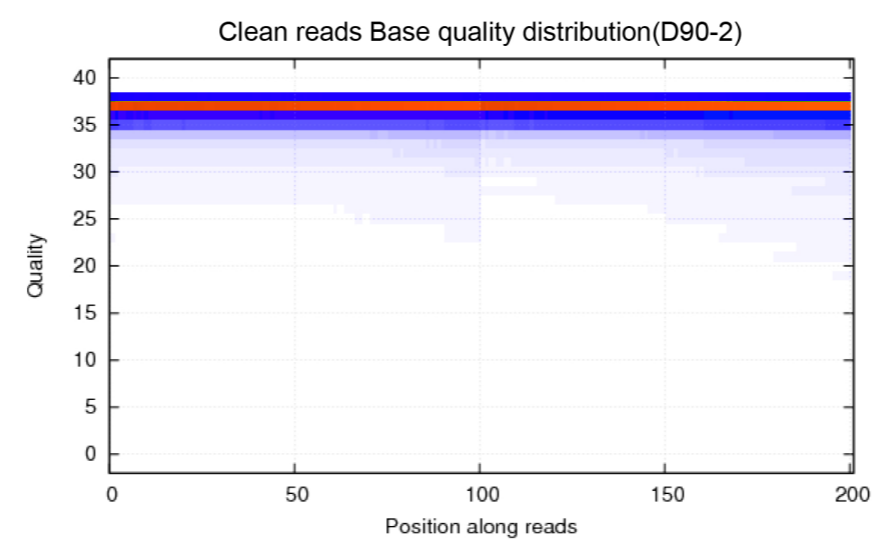

F

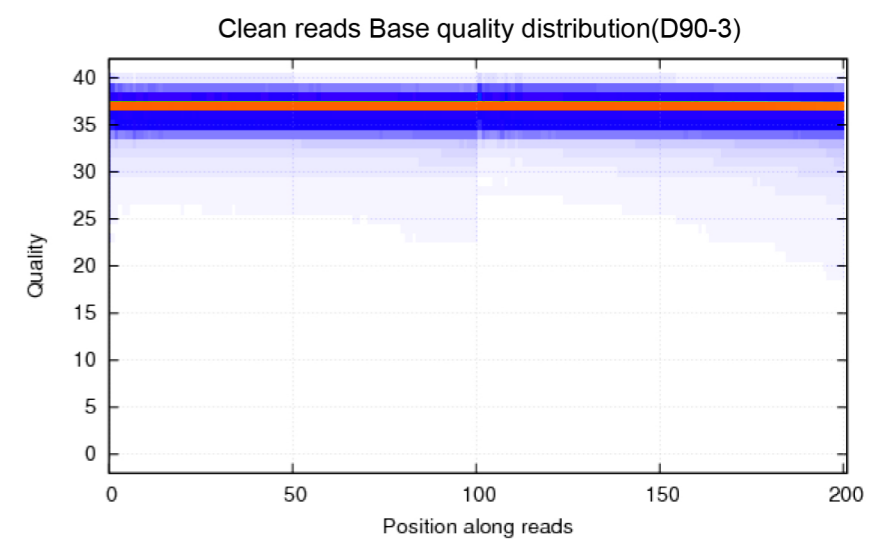

G

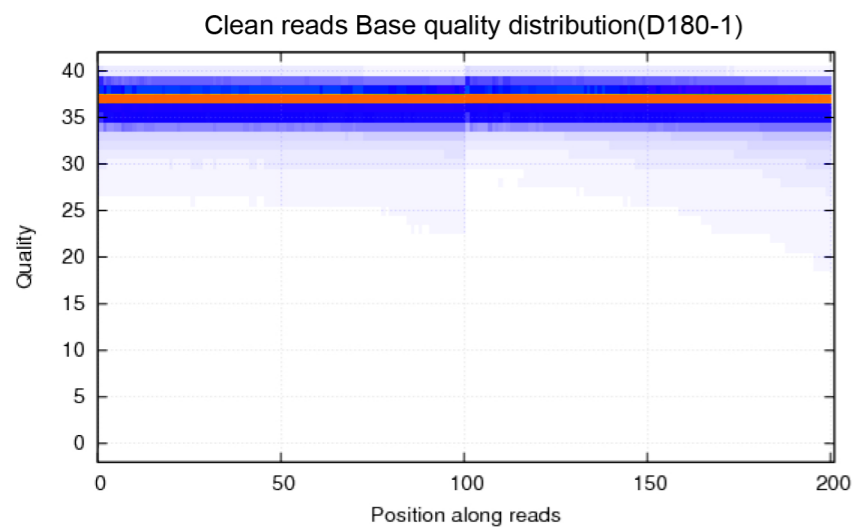

H

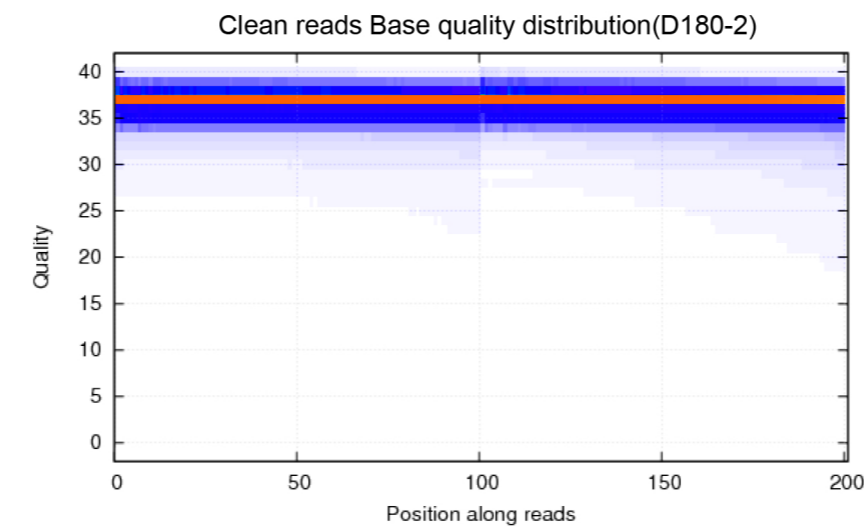

I

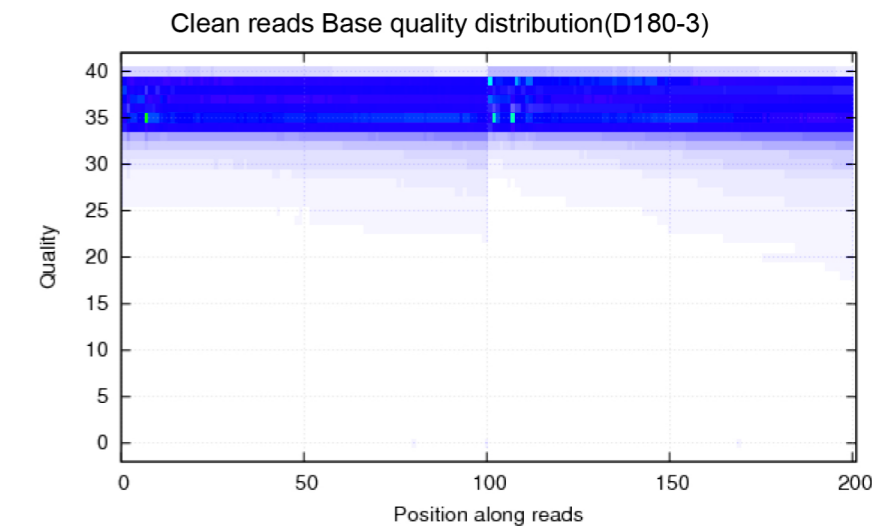

J

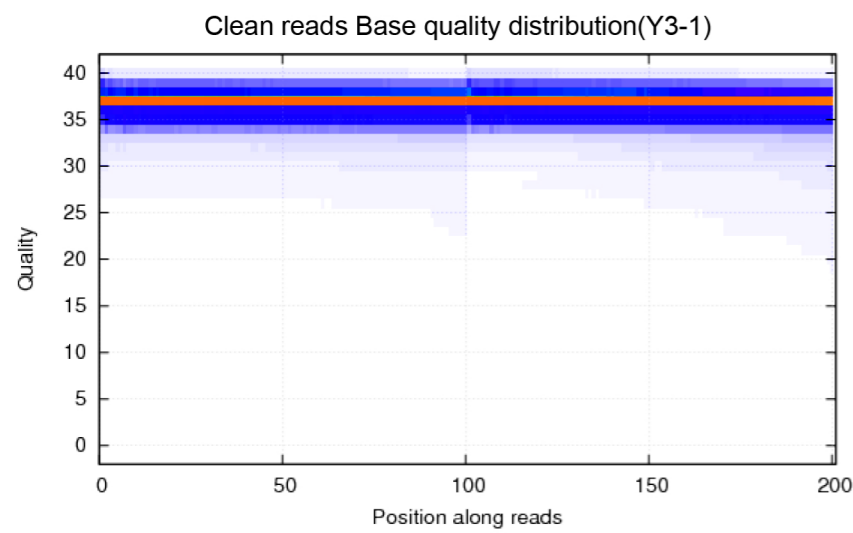

K

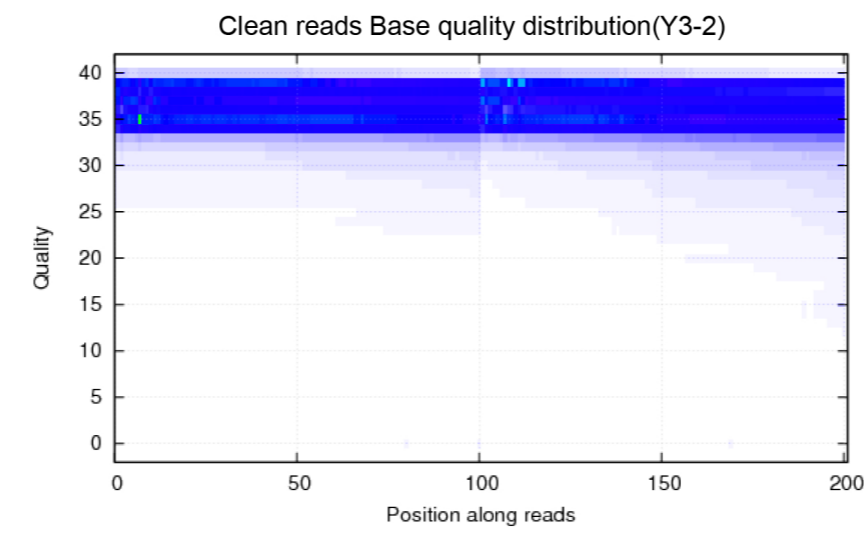

L

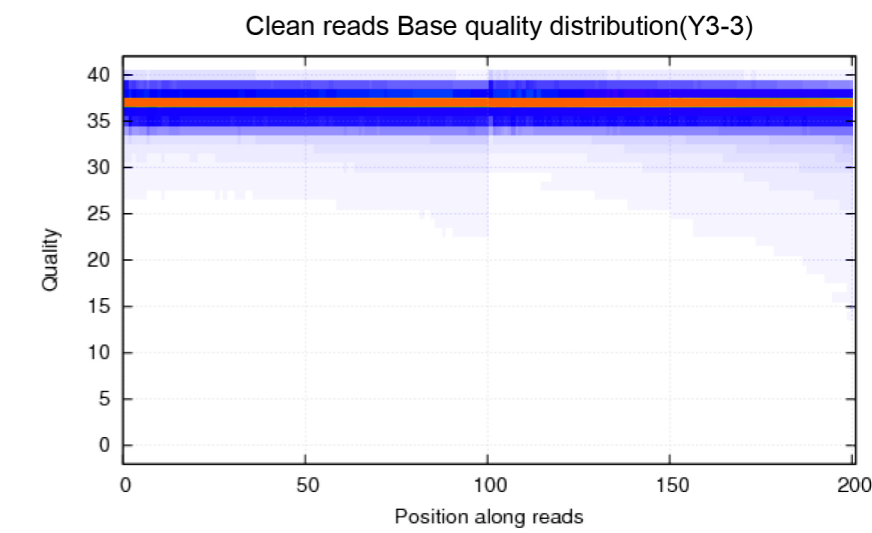

Supplement: Supplemental Information 3 [file peerj-11-15955-s003.pdf]

A

D180 / D90

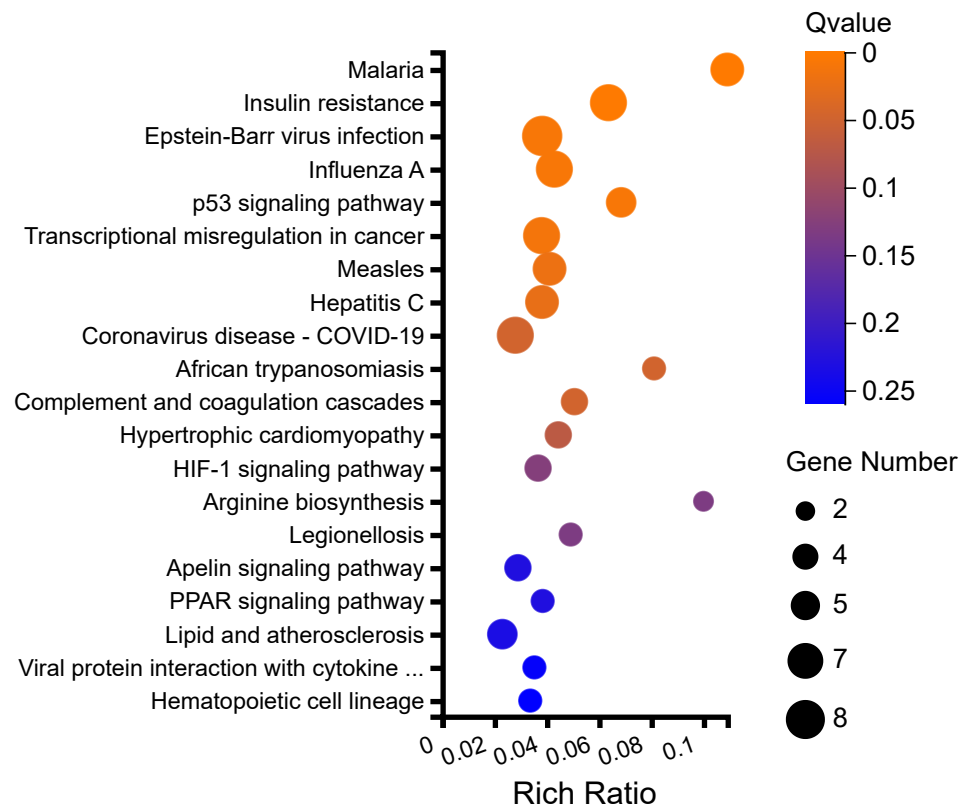

B

Y3 / D180

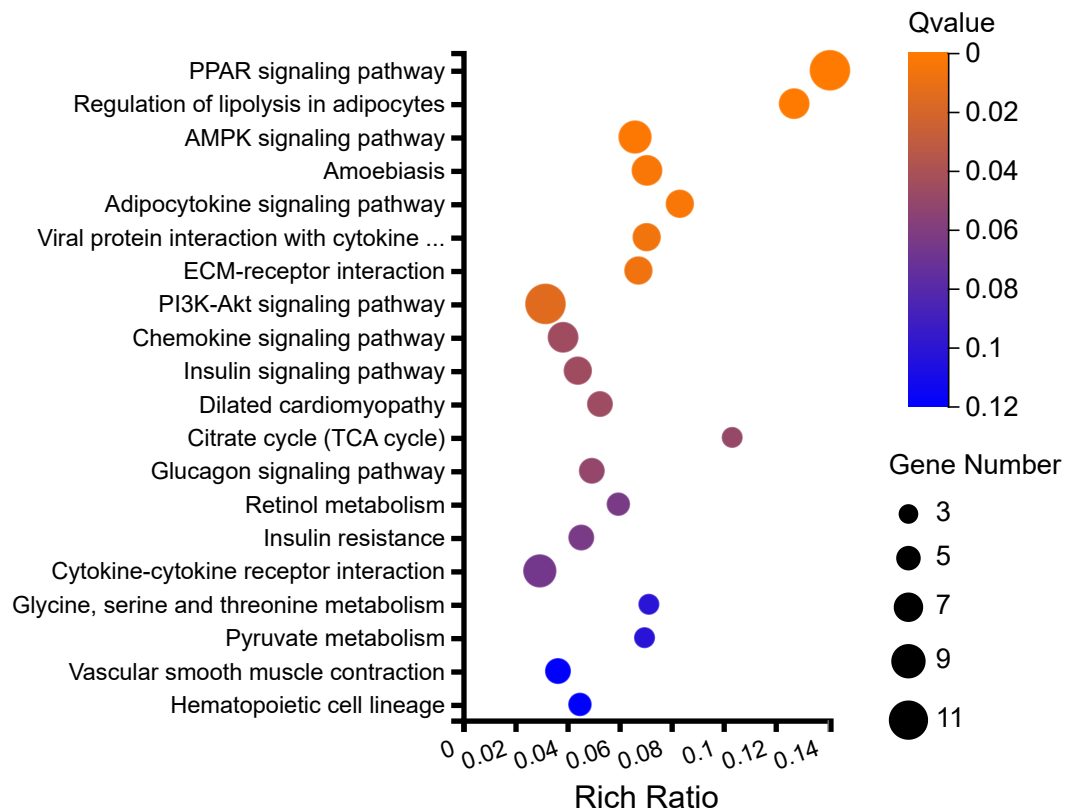

Supplement: Supplemental Information 4 [file peerj-11-15955-s004.pdf]

A

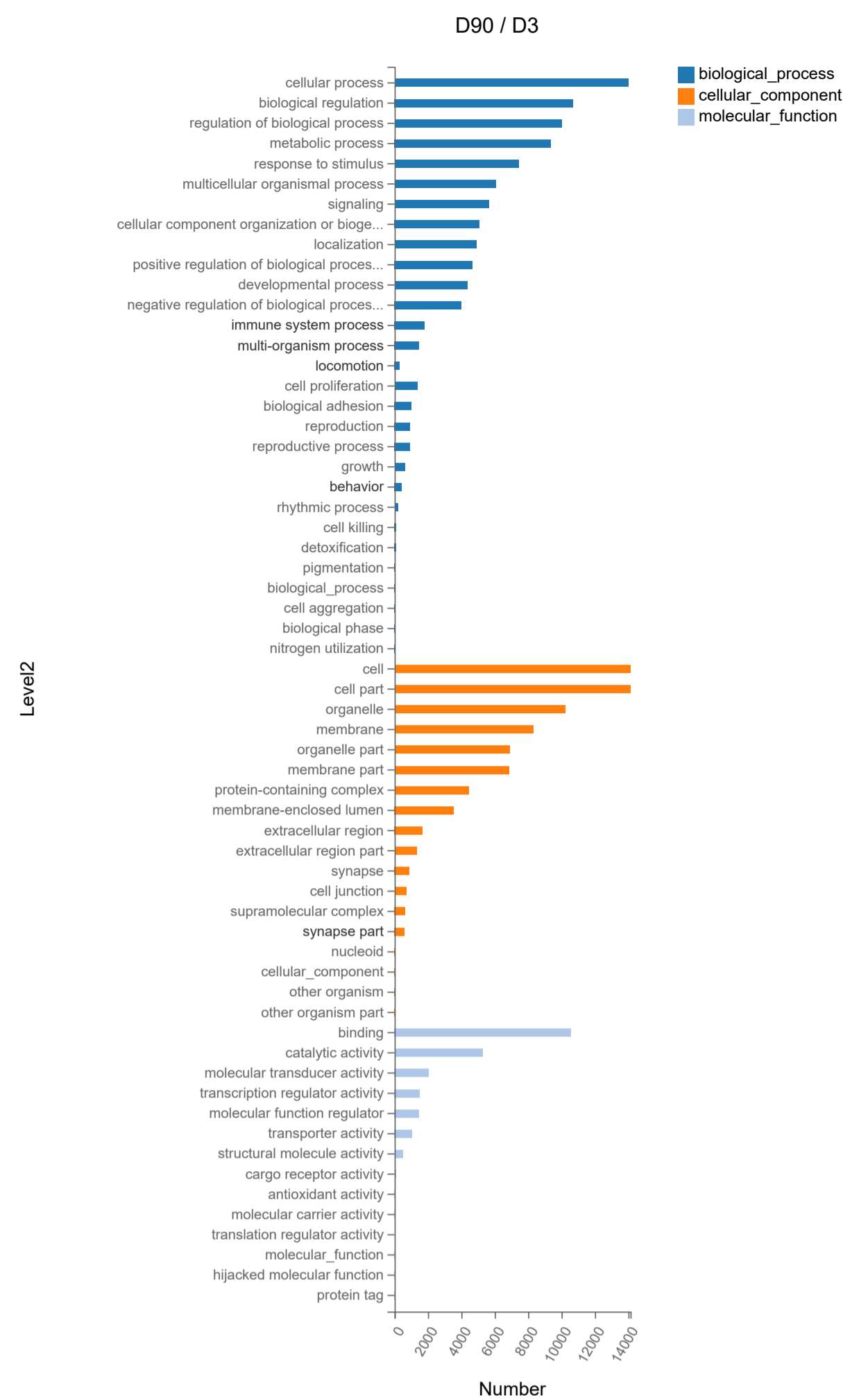

B

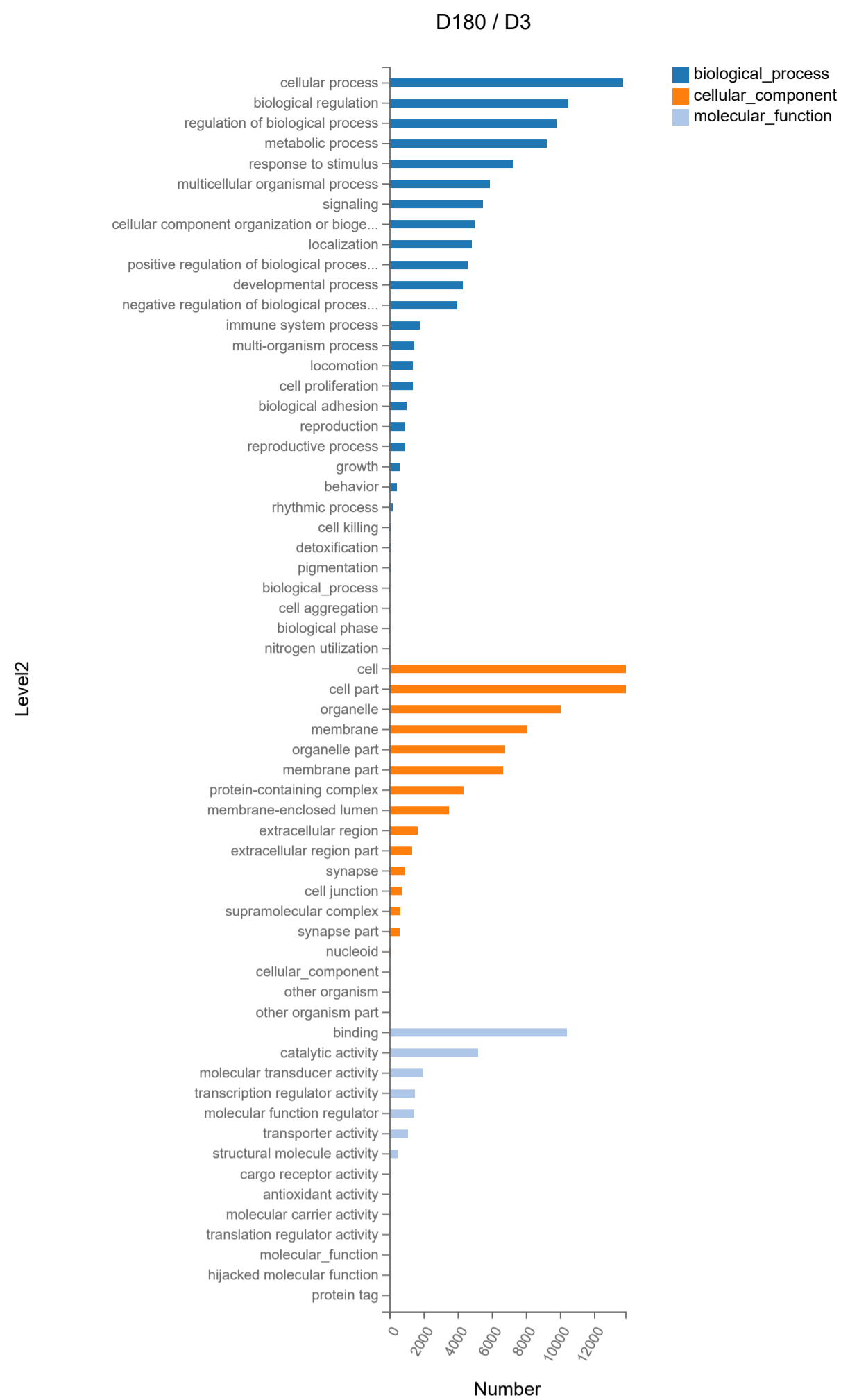

C

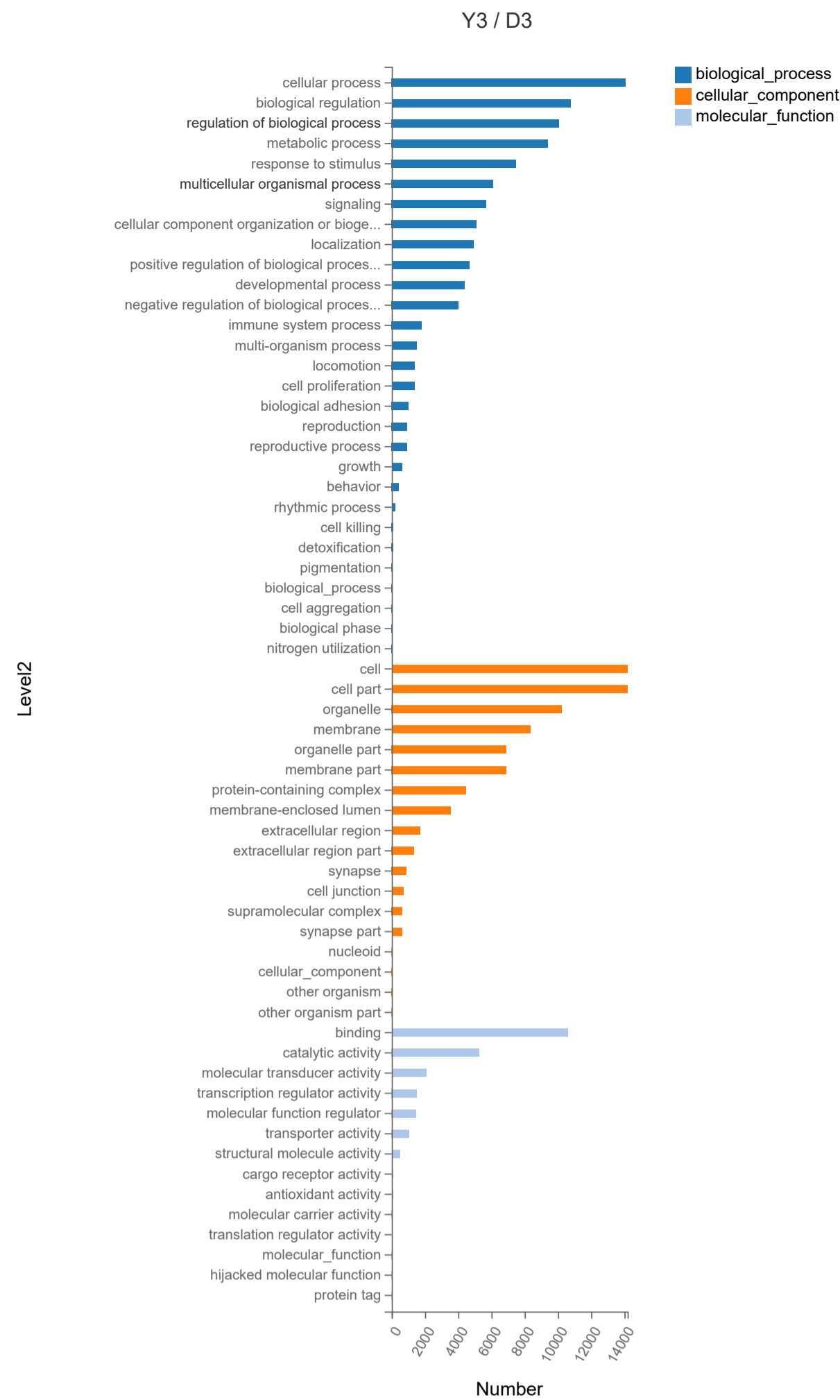

D

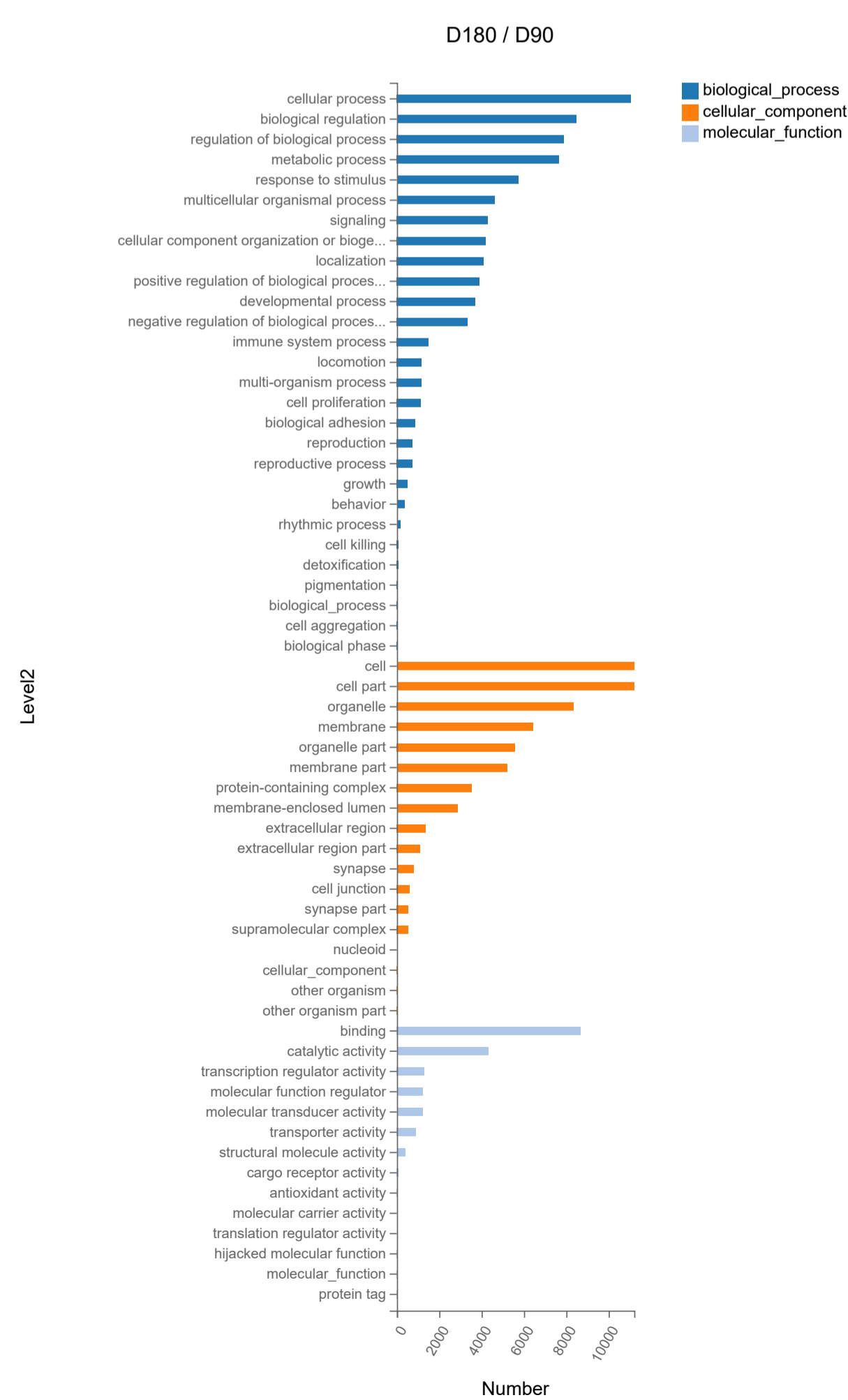

E

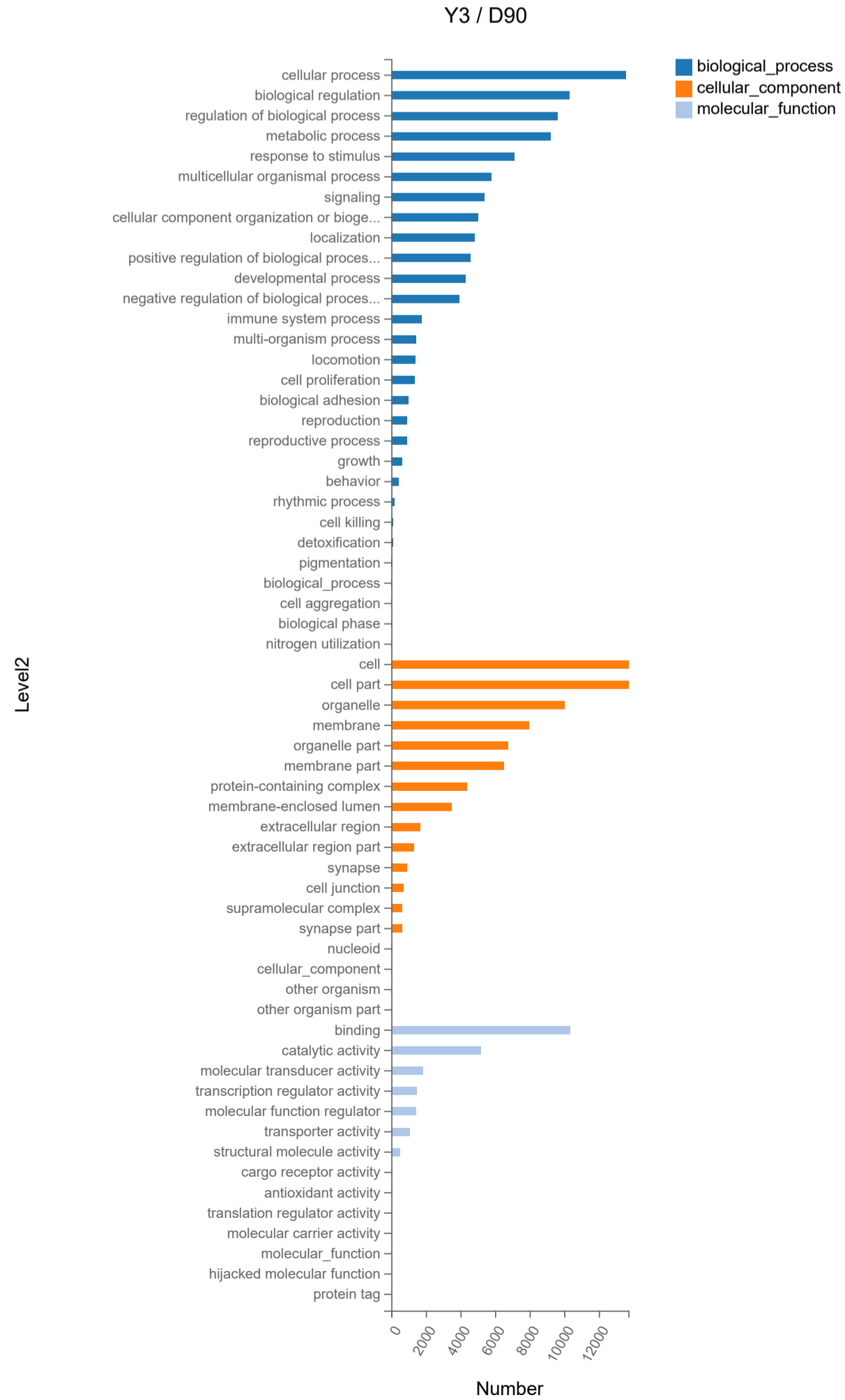

F

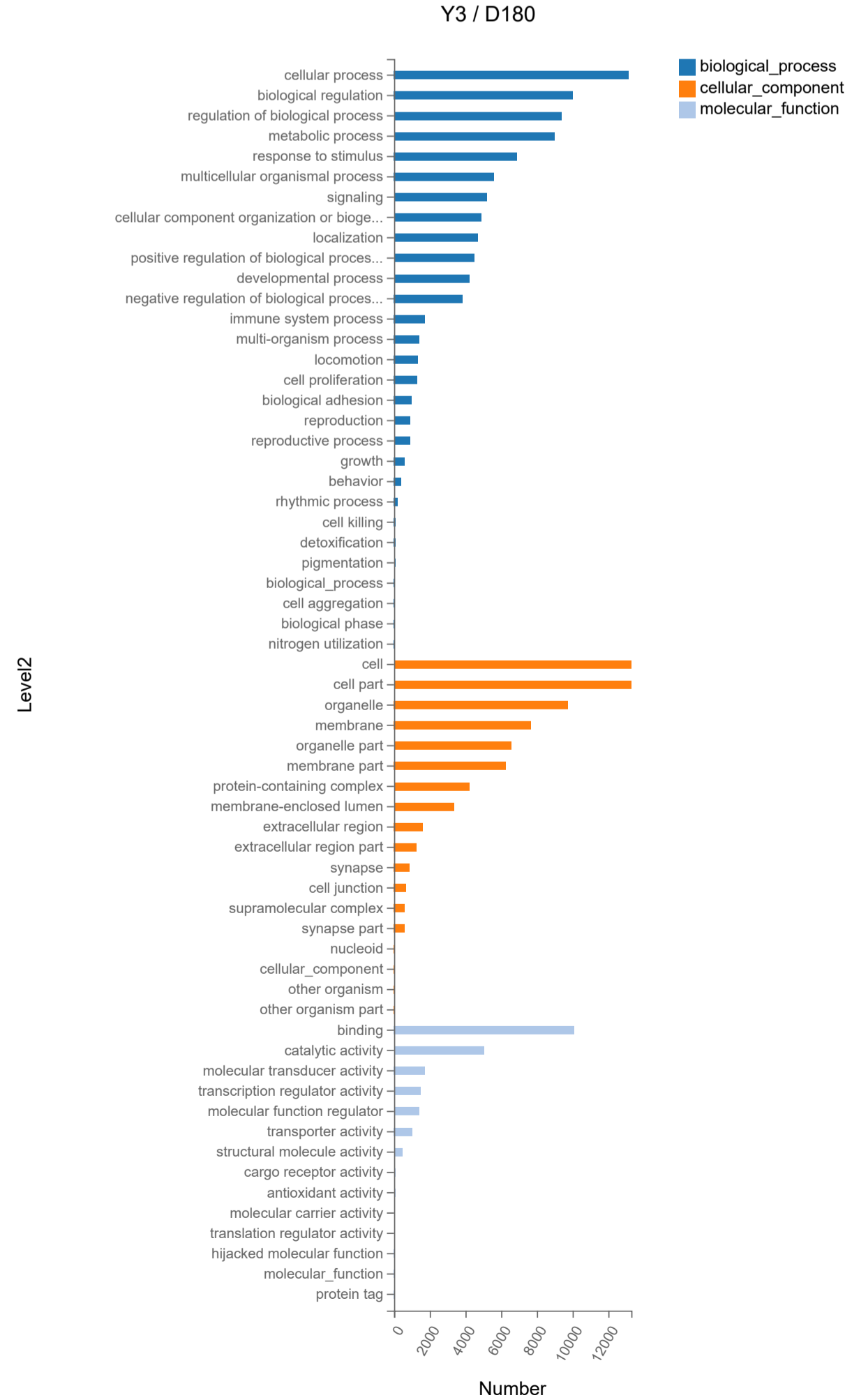

Supplement: Supplemental Information 5 [file peerj-11-15955-s005.pdf]

A

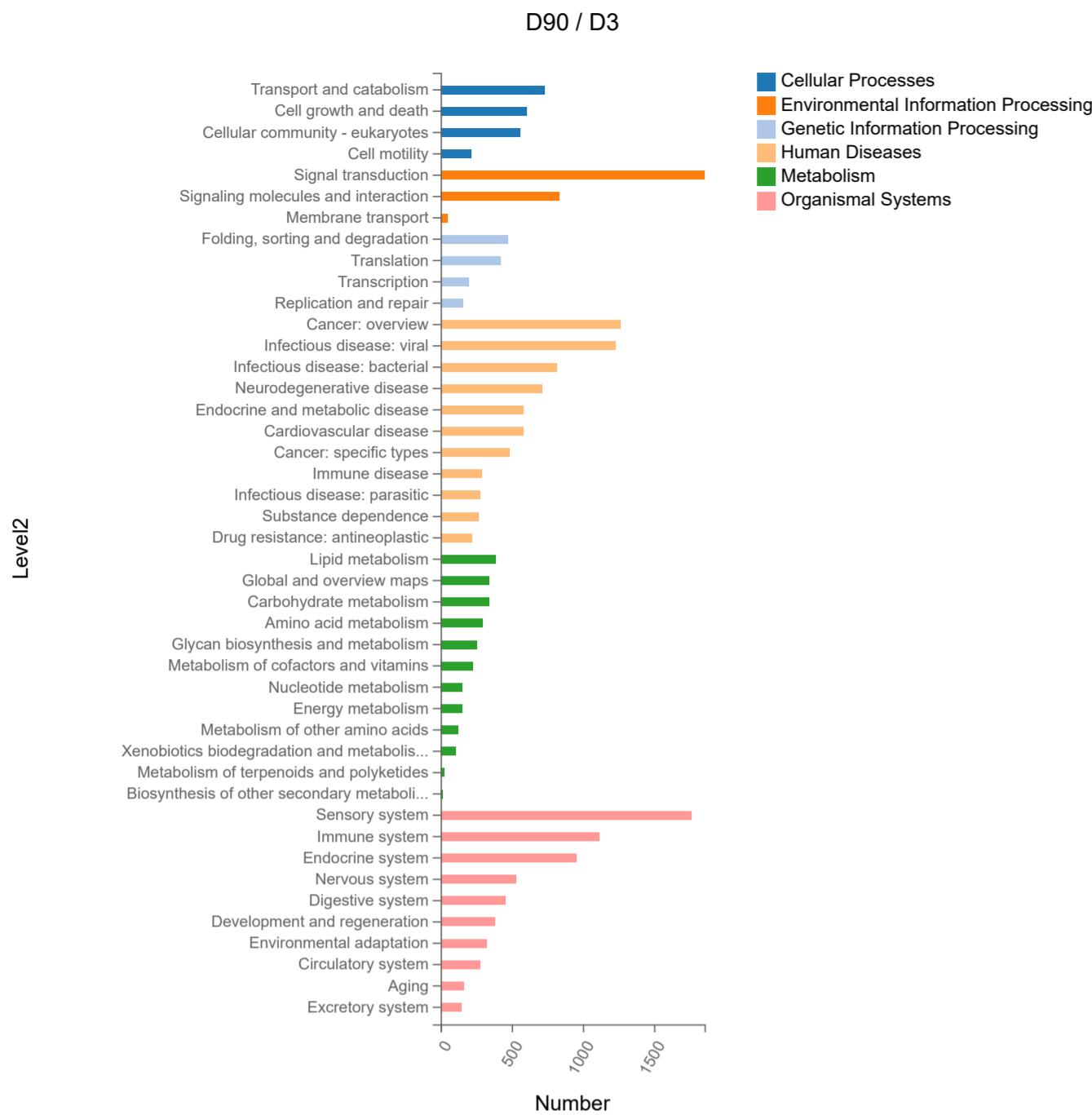

B

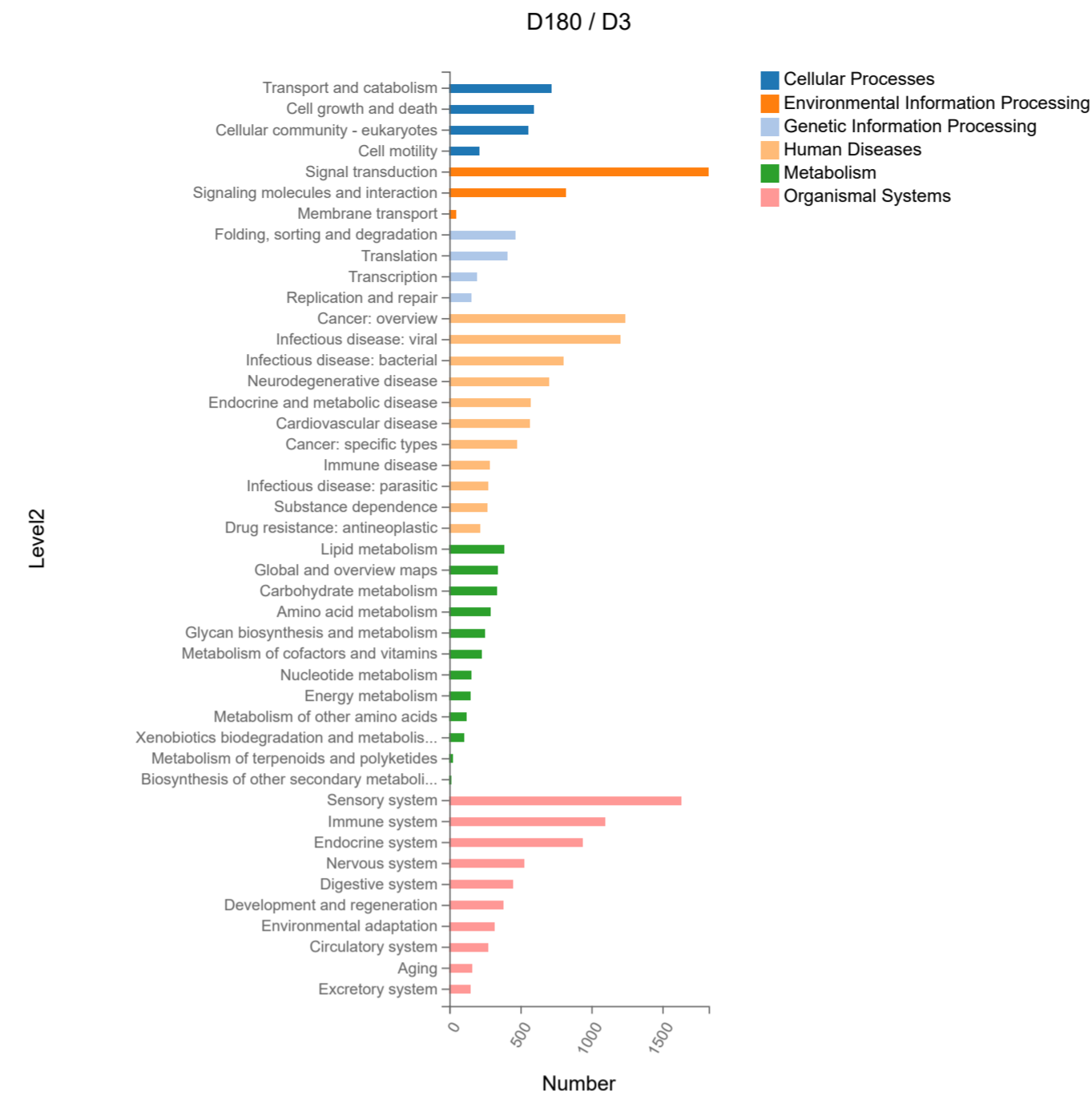

C

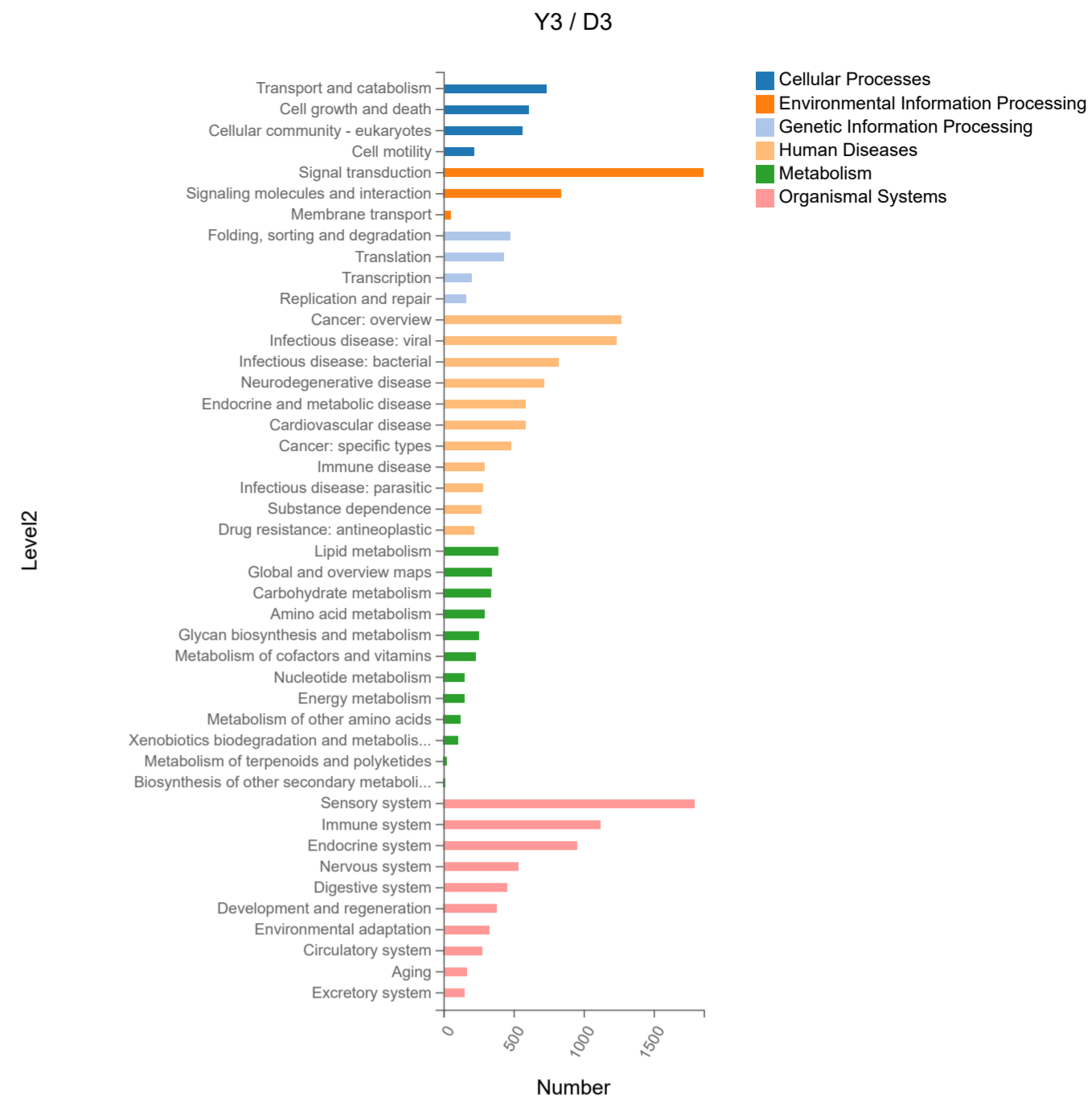

D

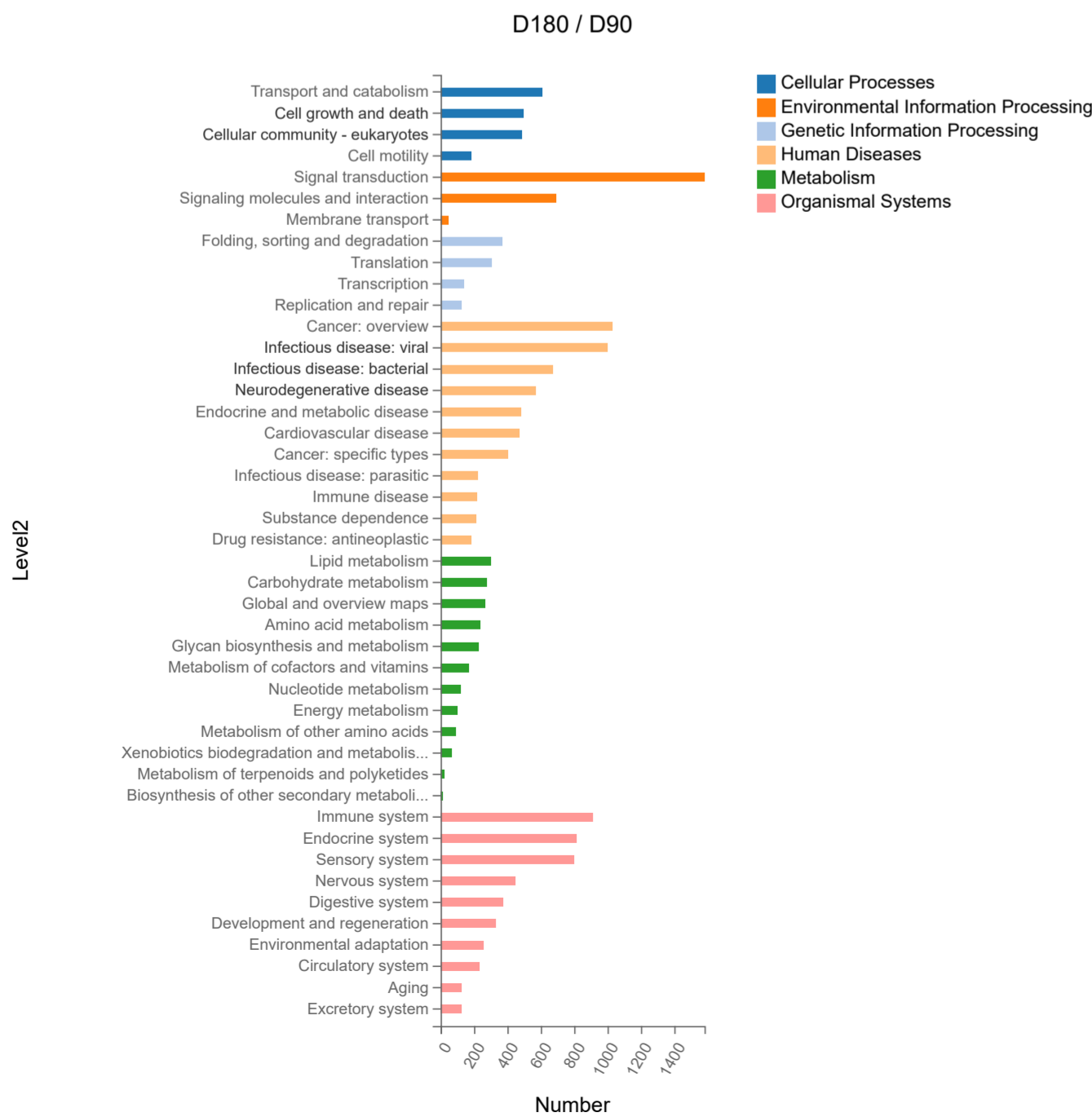

E

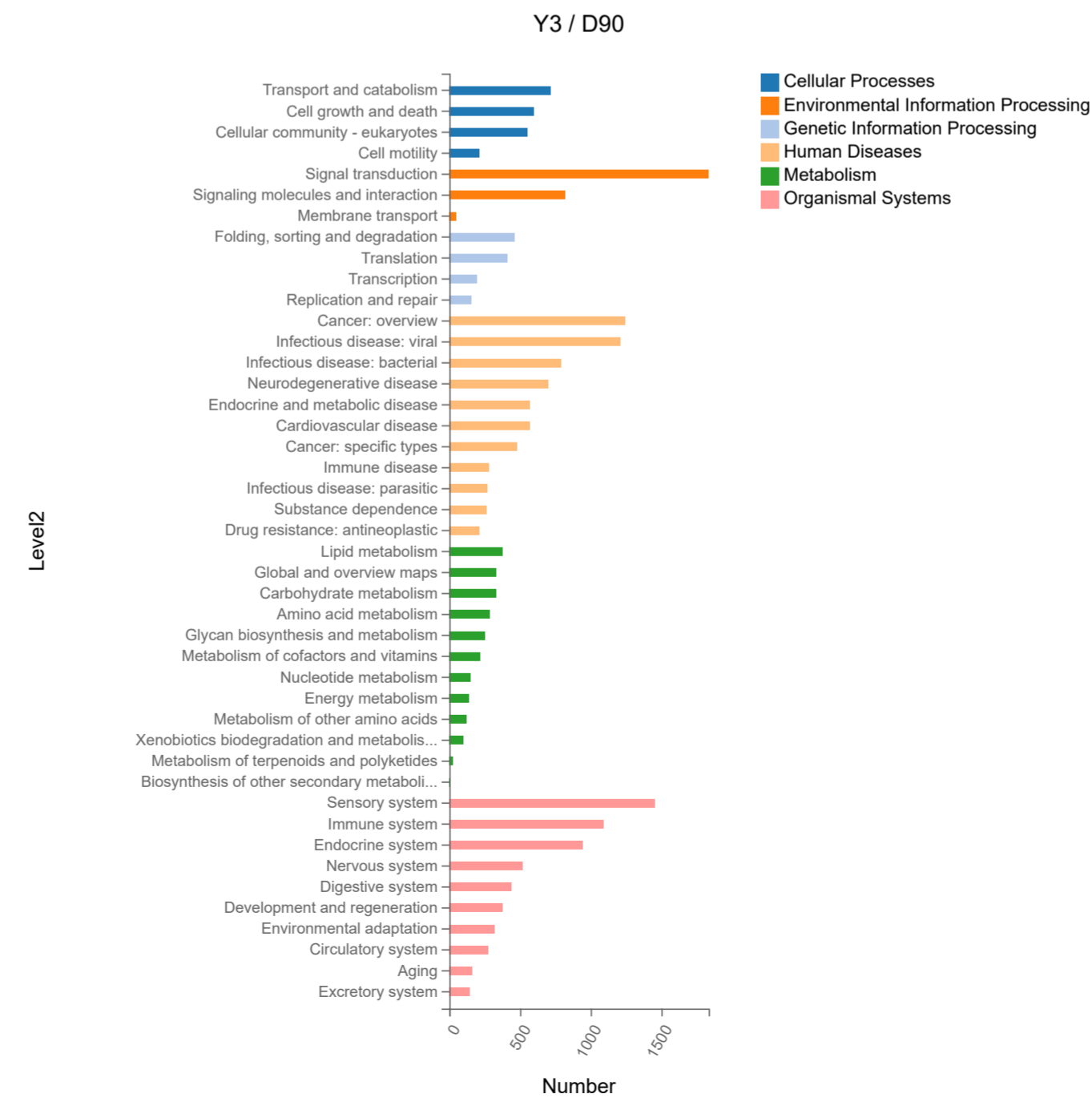

F

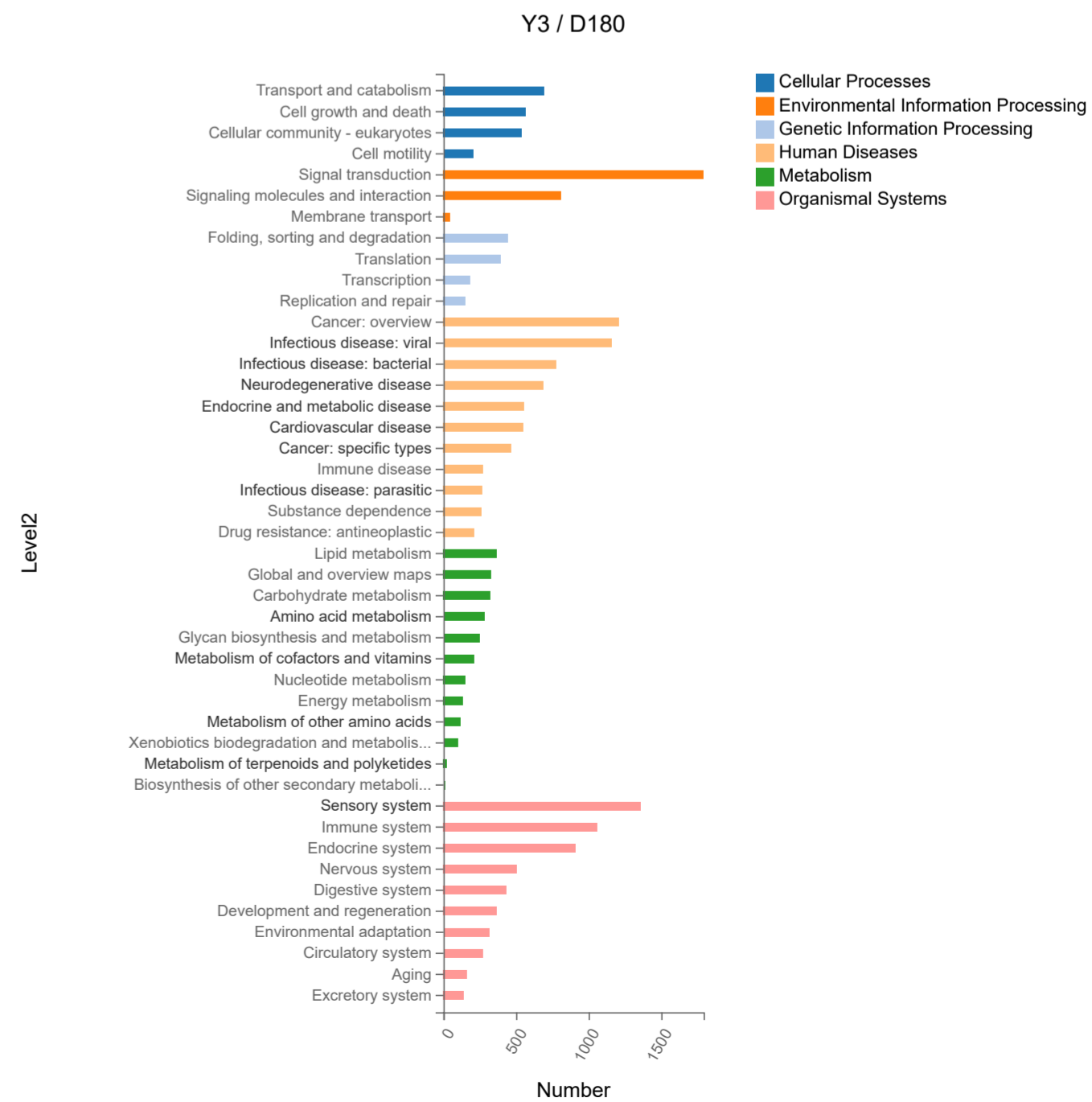

Supplement: Supplemental Information 6 [file peerj-11-15955-s006.pdf]

A

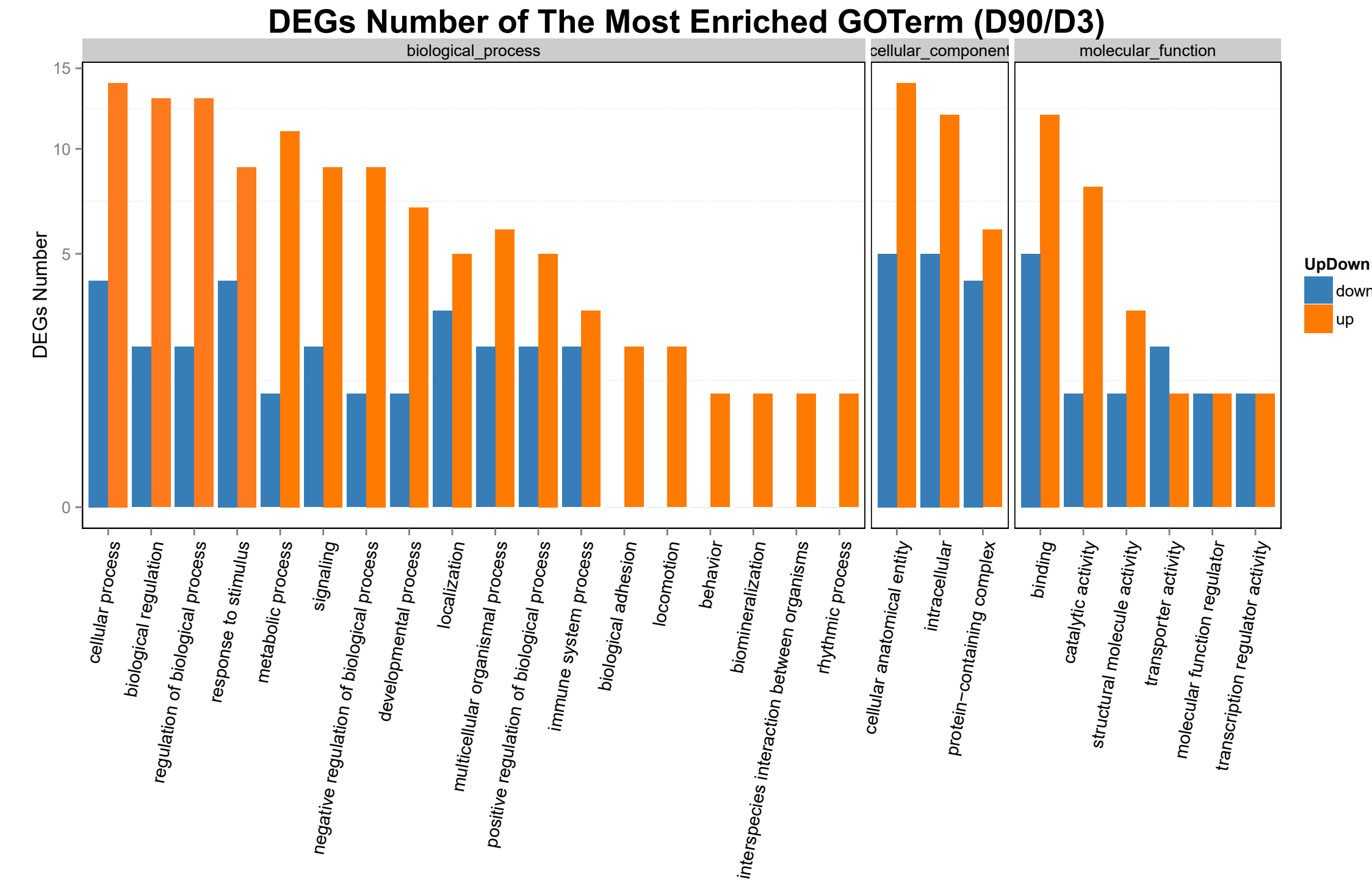

B

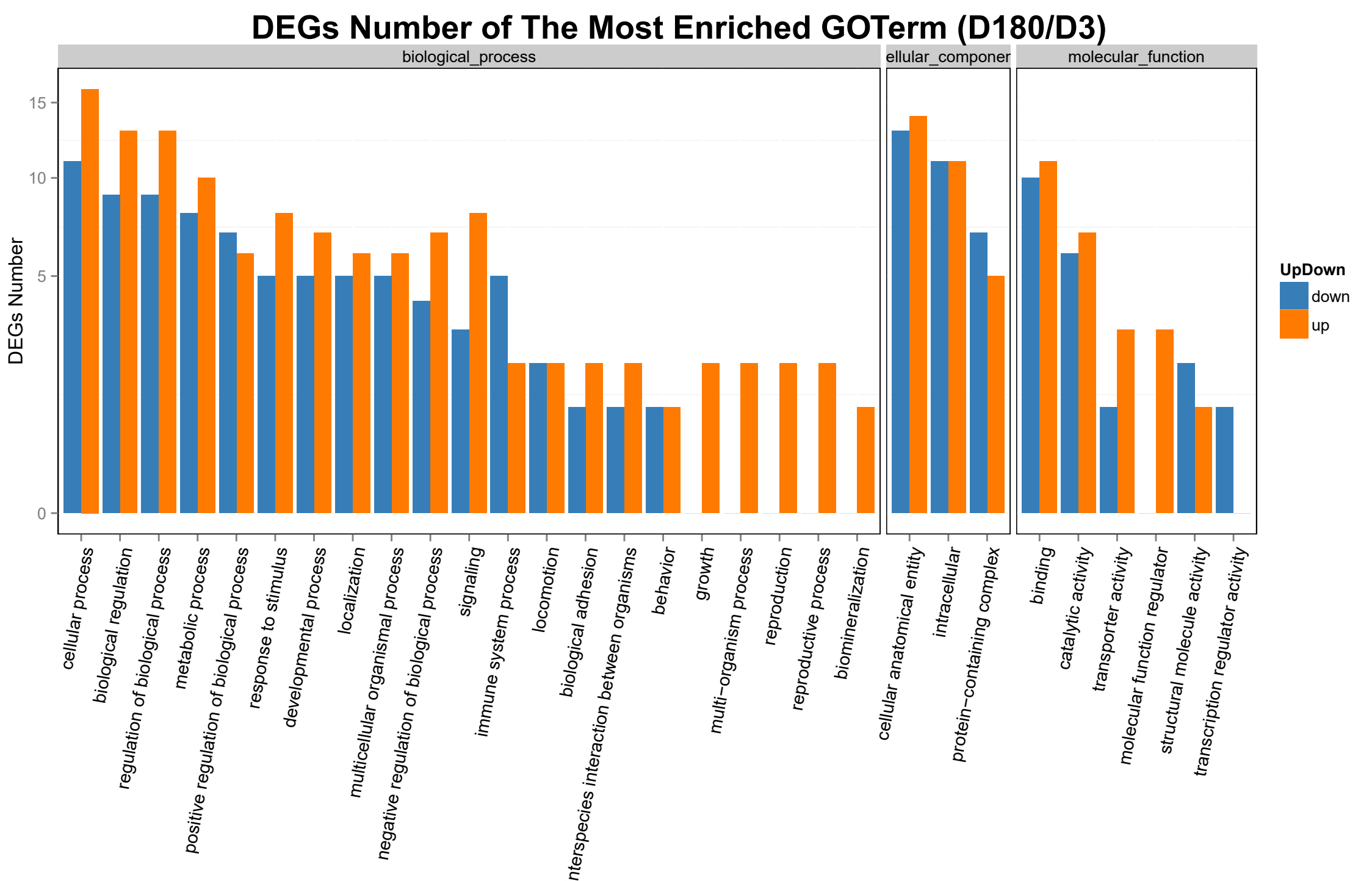

C

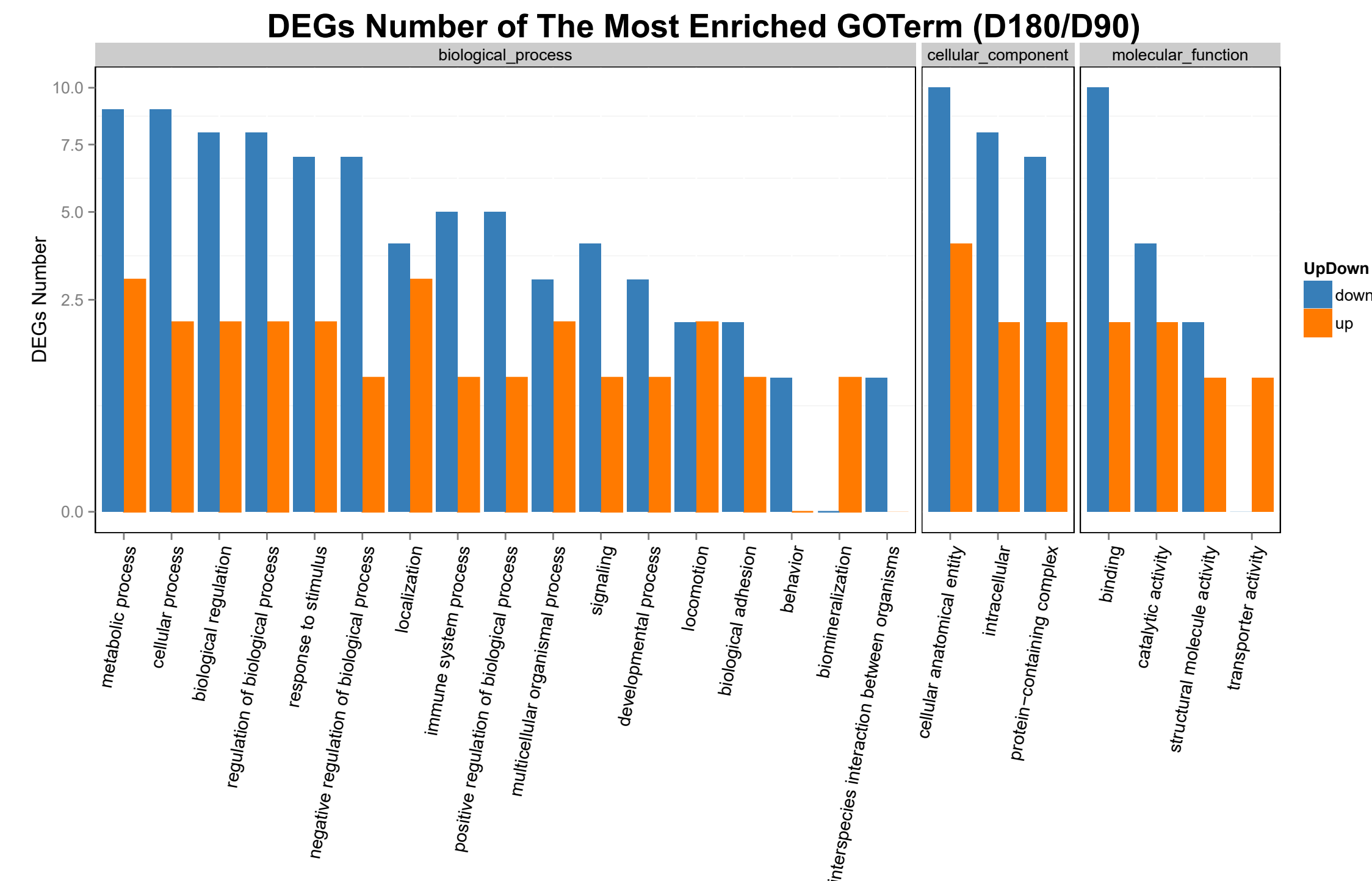

D

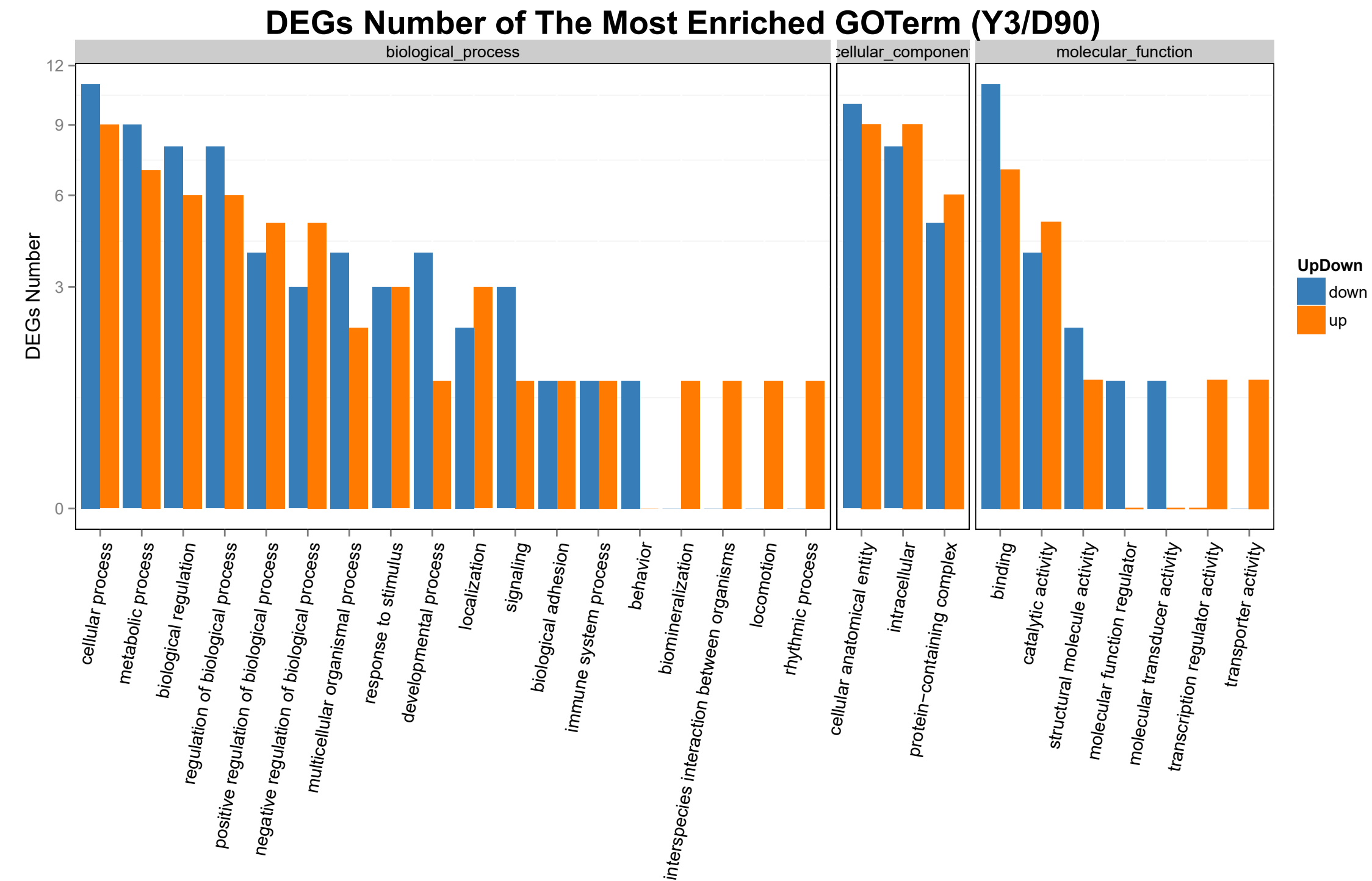

E

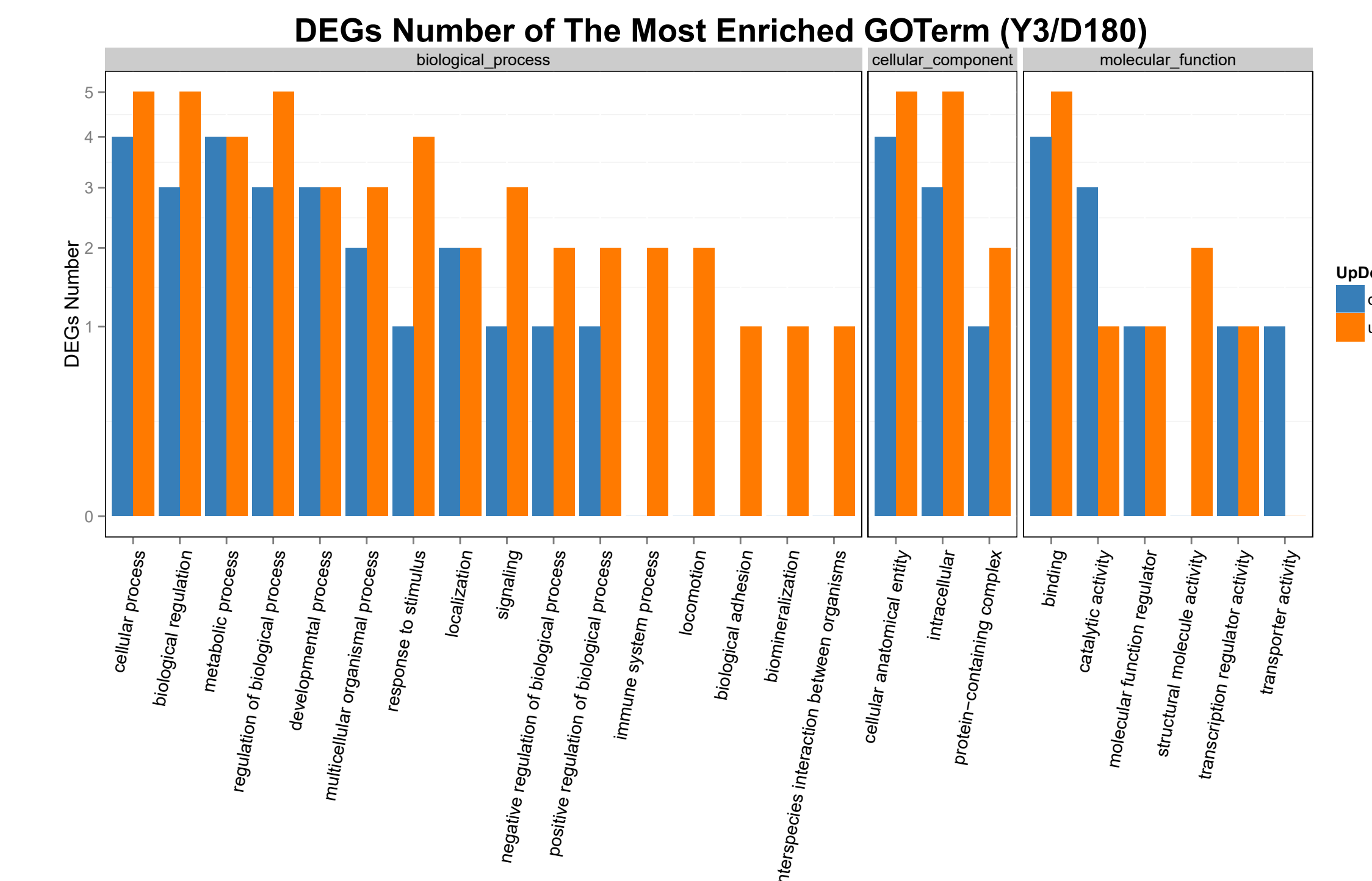

Supplement: Supplemental Information 7 [file peerj-11-15955-s007.pdf]

A

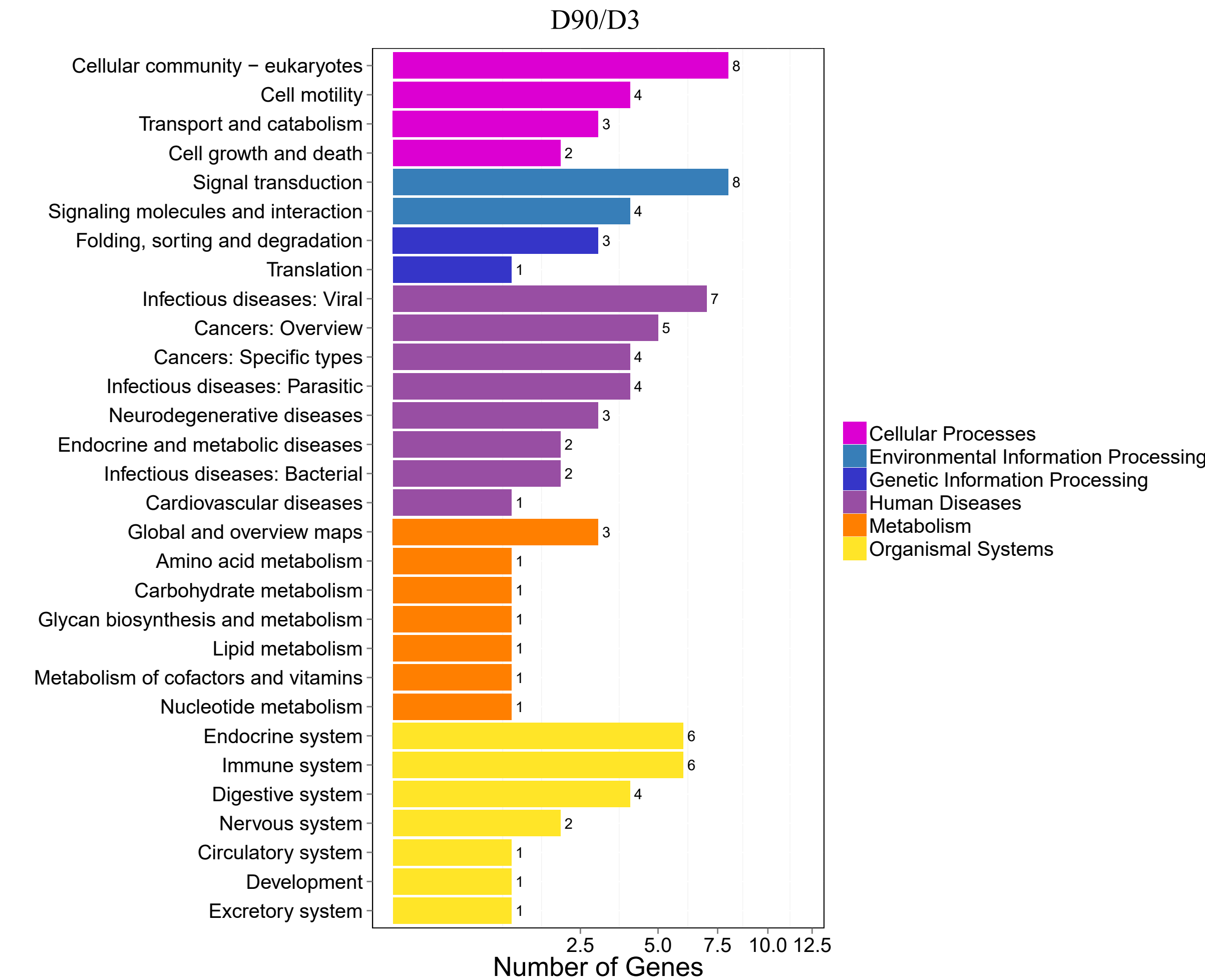

B

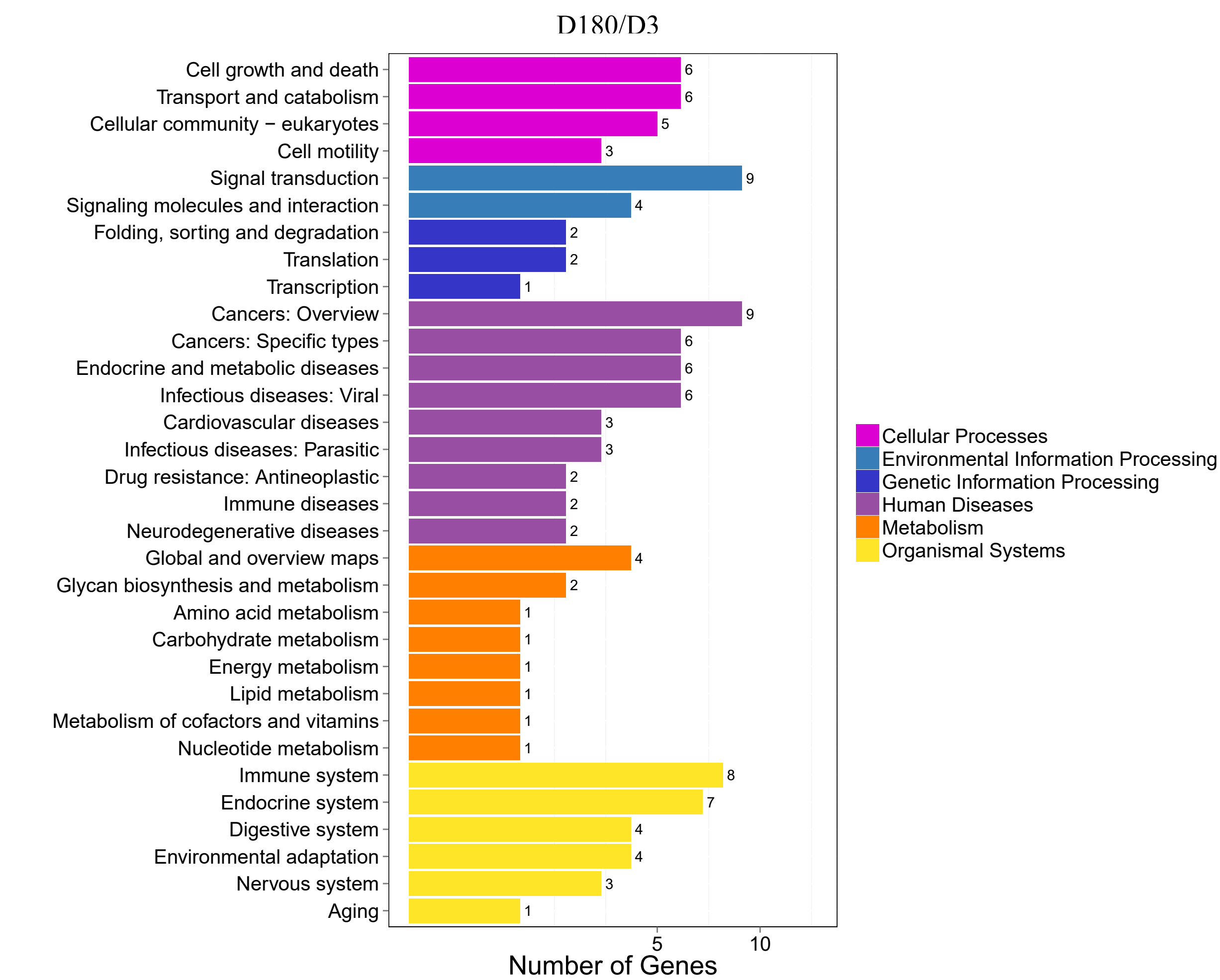

C

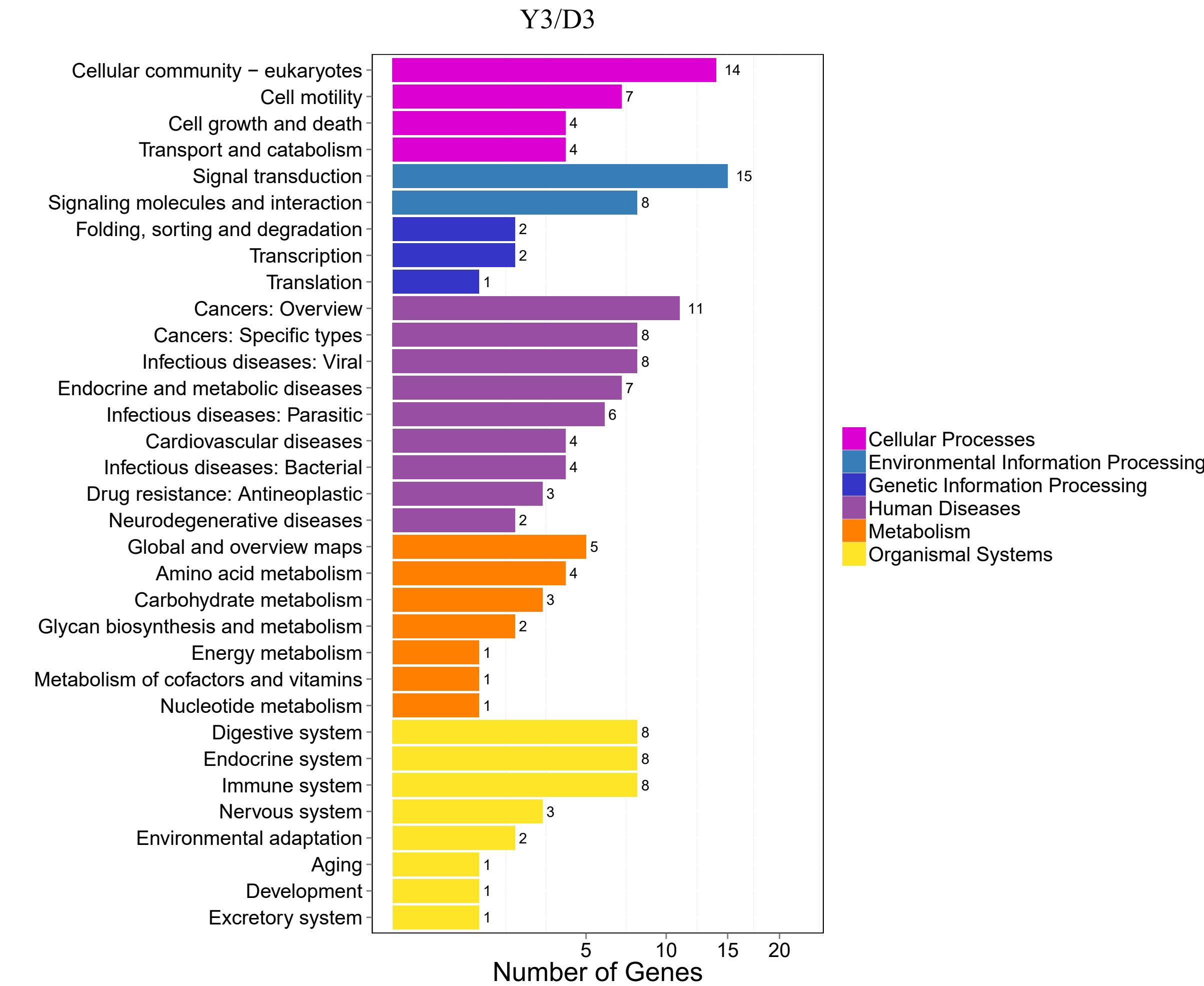

D

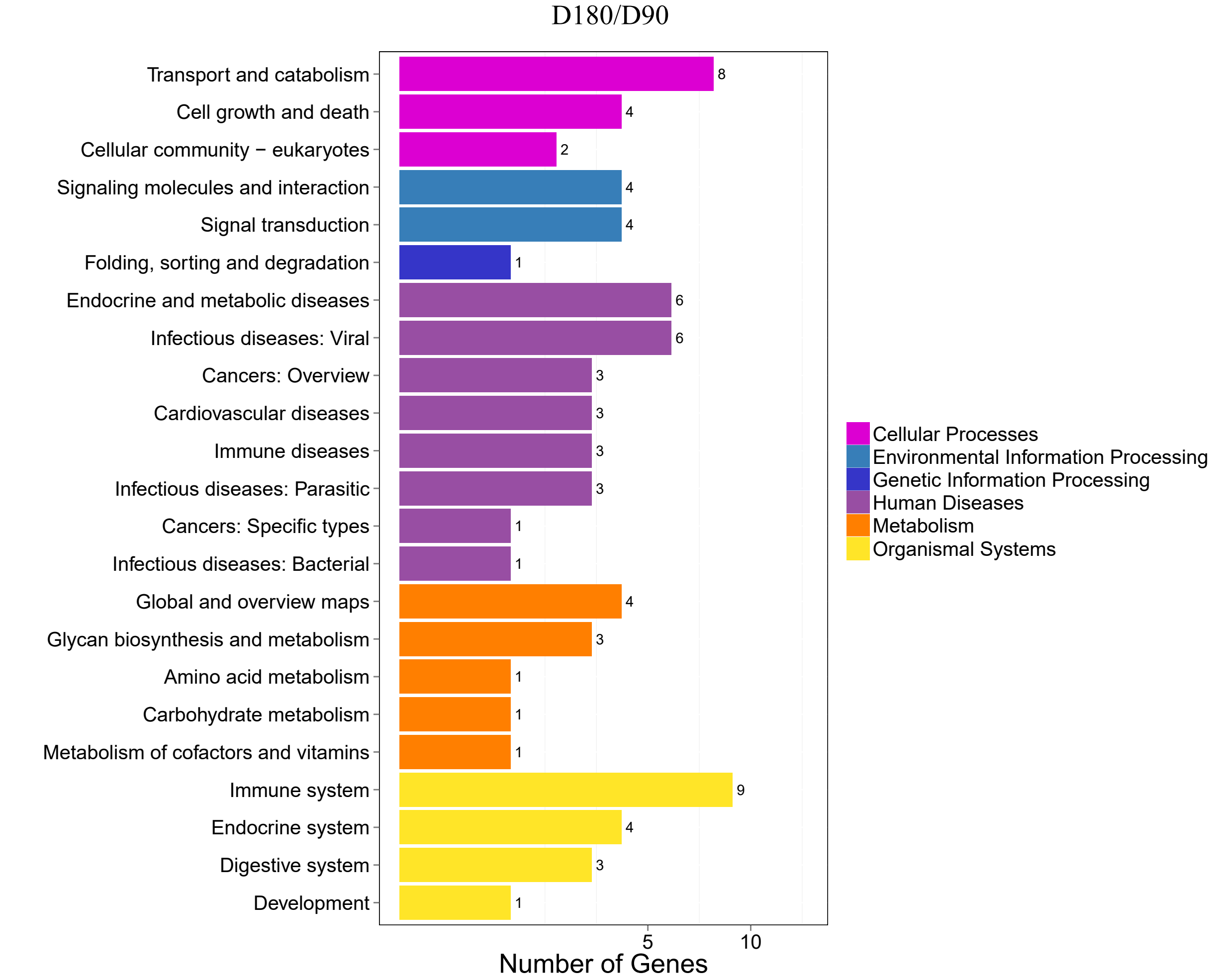

E

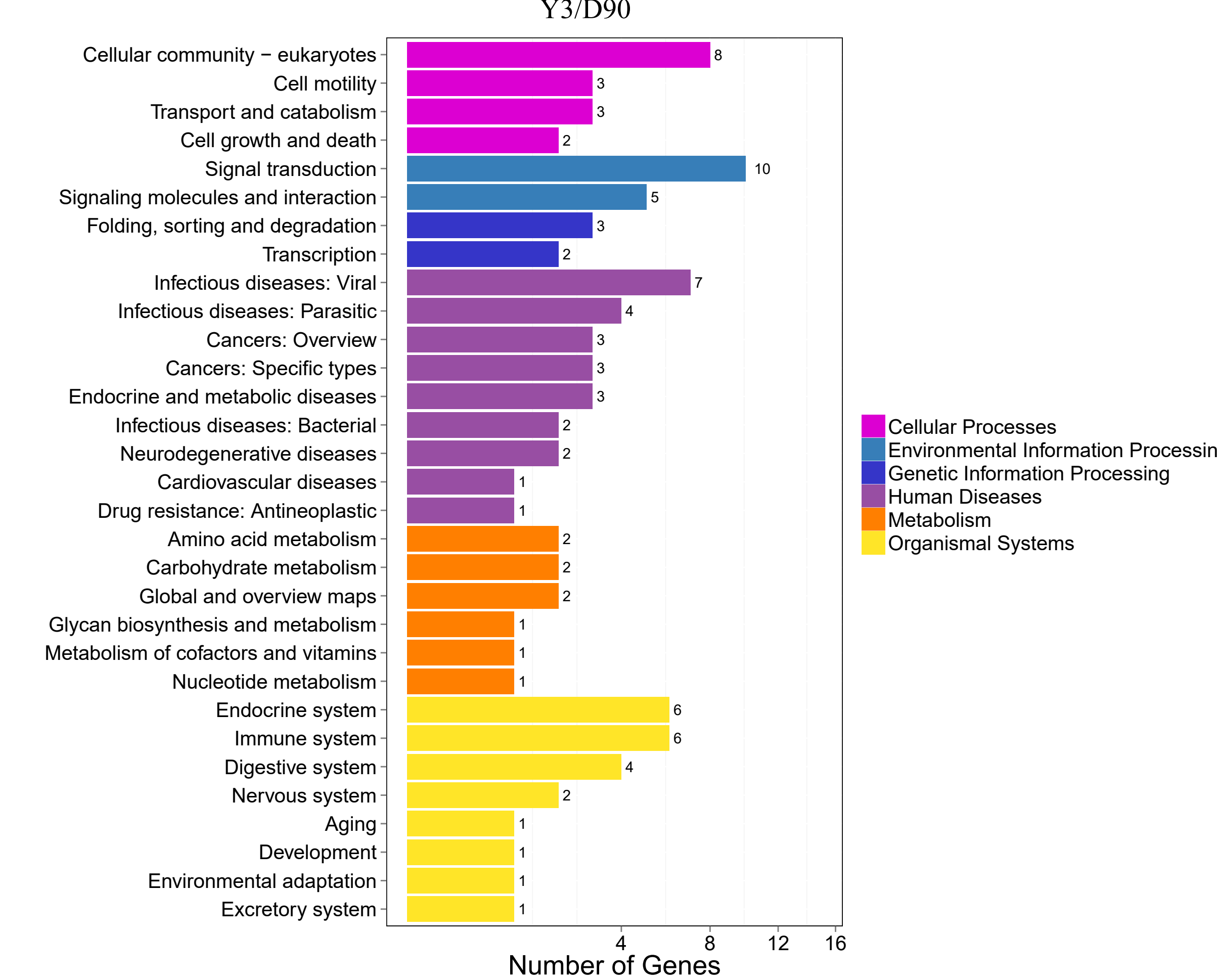

F

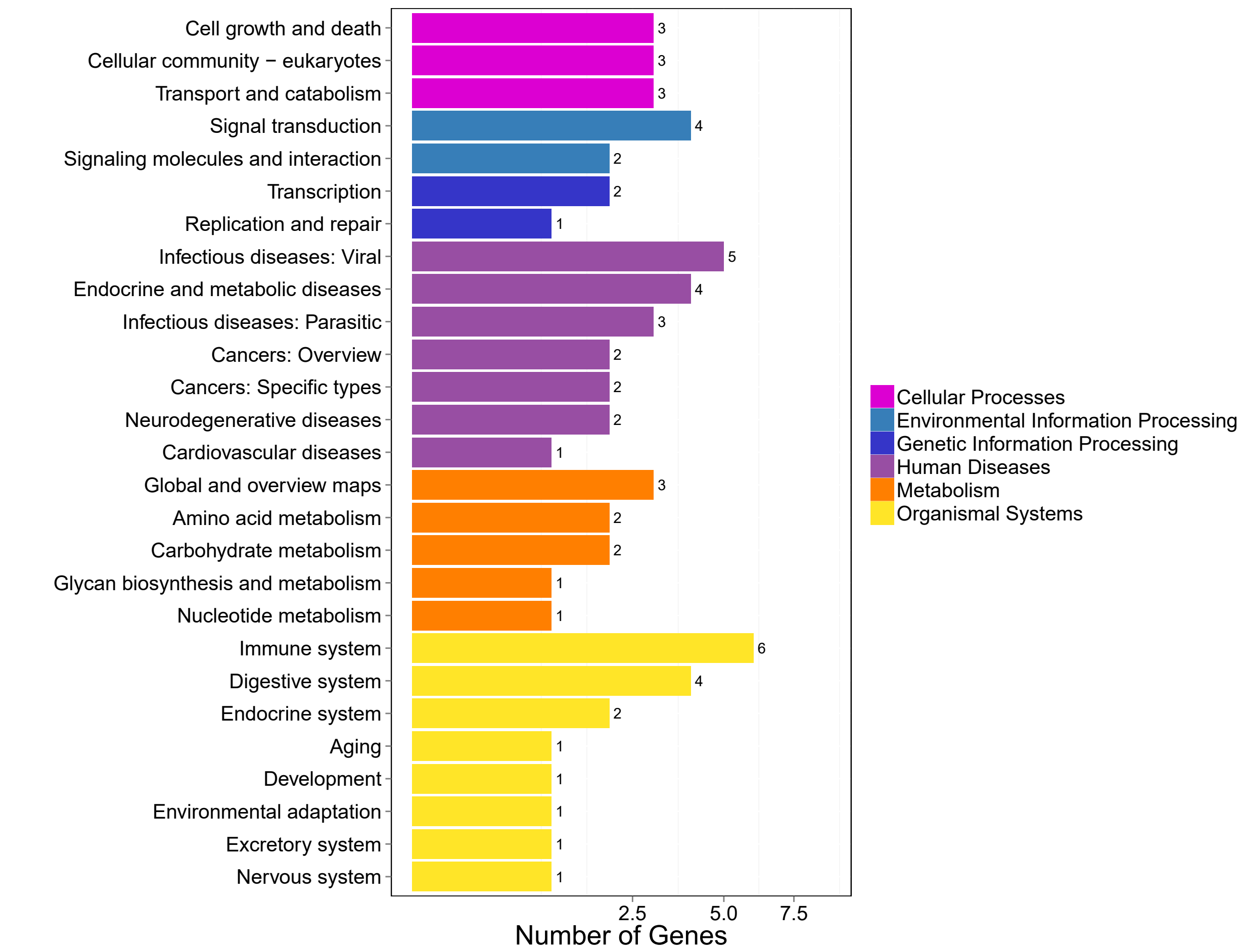

Supplement: Supplemental Information 8 [file peerj-11-15955-s008.pdf]
