# Supplementary material for: Comprehensive transcriptional analysis of pig facial skin development
Source: PeerJ. 2023 Aug 28;11:e15955. doi: 10.7717/peerj.15955 (PMC10470455; doi:10.7717/peerj.15955)
Supplement: Supplemental Information 9 [file peerj-11-15955-s009.pdf]

Table S1 The primer of RNAs

RNAs	Type	Primer(5′–3′)
GAPDH	-	F: GTGACGTTGACATCCGTAAAGA<br>R: GCCGGACTCATCGTACTCC
U6	-	F: CTCGCTTCGGCAGCACA<br>R: AACGCTTCACGAATTTGCGT
CTSK	mRNA	F: TGGATAATTAAAAACAGCTGGGGA<br>R: TGAGGTTGCCTGGCTGAAG
ELN	mRNA	F: CCAAAGCCCAGTTCCAGGC<br>R: CTTTAGCTGCGGCCAGGG
MEG3	lncRNA	F: CCAGCCTACGAAGAAAGCCA<br>R: CGGCATTCGGCGATAACAAG
URS0001952842	lncRNA	F: ACGGCGGCAATATCAAGTCA<br>R: TTGCCCATGCTTGGTTGAGT
ssc-miR-30b-5p	miRNA	F: TGTAAACATCCTACACTCAGC<br>R: CAGGTCCAGTTTTTTTTTTTTTT
novel-circ-008475	circRNA	F: TGGGTTAATGGAAACAGCGAAA<br>R: CTCAGCGGTCGCATTTTCCTAA

GAPDH, the internal reference for mRNA, lncRNA, and circRNA. U6, the internal reference for miRNA.
